# Prediction of antimicrobial minimum inhibitory concentration from bacterial genomes using a scalable and interpretable machine learning approach

**DOI:** 10.1038/s44259-026-00217-4

**Published:** 2026-05-26

**Authors:** Alessandro Gerada, Yinzheng Zhong, Nicholas Harper, Anoop Velluva, Nada Reza, Vineet Dubey, Alex Howard, Peter L. Green, Steve Paterson, William Hope

**Affiliations:** 1 Department of Clinical Pharmacology and Therapeutics, Antimicrobial Pharmacodynamics and Therapeutics, Institute of Systems, Molecular & Integrative Biology, University of Liverpool, Liverpool, UK; 2 Department of Infection and Immunity, Liverpool Clinical Laboratories, Clinical Support Services Building (CSSB), Liverpool University Hospitals NHS Foundation Trust, Liverpool, UK; 3 School of Engineering, University of Liverpool, Liverpool, UK; 4 Institute of Infection, Veterinary and Ecological Sciences, University of Liverpool, Liverpool, UK

**Keywords:** Computational biology and bioinformatics, Microbiology

## Abstract

Although machine learning models can predict antimicrobial susceptibility from bacterial whole genome sequencing (WGS), state-of-the-art approaches are computationally demanding or dependent on knowledge of genetic resistance determinants. Here, we describe an efficient data-driven approach to predicting minimum inhibitory concentration (MIC) by progressively extending and refining predictive genome segments, independent of prior knowledge of resistance determinants. Resultant models had high interpretability — known and potentially novel resistance determinants were captured. Using 762 clinical *E. coli* strains, 71.6% of predictions were within one dilution of the measured MIC. Models trained with this algorithm generalised better onto external data (F1 score = 0.85) compared with alternative models trained on annotated resistance determinants (F1 = 0.82) or *k*-mer counts (F1 = 0.74). Computational demands were low (RAM usage 23.6GB vs 38.8GB for *k*-mer model). These advantages represent an important advance in predicting antimicrobial susceptibility from WGS, with potential applications for clinical diagnostics, drug development, and surveillance.

## Introduction

Antimicrobial susceptibility testing (AST) underpins the clinical care of patients with infection, antimicrobial surveillance, and the development of new antimicrobial agents. With the increasing availability of whole genome sequencing (WGS) data, there is a growing interest in predicting antimicrobial susceptibility directly from genomic data^1^. The use of WGS may help address some of the limitations of routine AST, such as inconsistencies between phenotypic methods and inter-laboratory variation, while providing the opportunity for rapid diagnostics^2^.

Although machine learning can predict antimicrobial susceptibility from genomic data, most approaches rely on known antimicrobial resistance (AMR) genetic determinants (e.g. *bla*_CTX-M_ for third-generation cephalosporin resistance) or single-nucleotide polymorphisms (SNPs) as model inputs, with binary (i.e. susceptible/resistant) phenotypes as model outputs^3,4^. The results of such models are promising, with high prediction accuracy reported in multiple studies^5,6^. Such models have also been shown to be generalisable across different bacterial species^7^. The strengths of using AMR determinants as model inputs are that the predictions are based on known mechanisms of AMR; therefore, the interpretability of resultant models is high. The main limitation of this mechanistically-informed approach is that it does not capture novel resistance mechanisms. An alternative approach is to use *k*-mer counts, which are *k*-length sub-strings of DNA sequences generated directly from assembled genomes, as model inputs^8–10^. While *k*-mer-based models do not require prior knowledge of AMR determinants, they are slow and memory-intensive to train, due to the high dimensionality of the data.

Although the binary classification of predictions (i.e. susceptible, ‘S’, or resistant, ‘R’) is sufficient for many applications, the MIC is a more complete measure of antimicrobial susceptibility that forms the basis of pharmacodynamic target attainment and has the benefit of facilitating more precise patient care. Additionally, the MIC is more applicable for AMR surveillance, since classification into ‘S’ or ‘R’ depends on breakpoints set by the European Committee on Antimicrobial Susceptibility Testing (EUCAST) and the Clinical and Laboratory Standards Institute (CLSI)^11,12^.

This study aimed to efficiently predict the MIC of *Escherichia coli* strains to commonly used antimicrobials from WGS, without a priori information on resistance determinants, while retaining model interpretability. We developed a novel algorithm, PGSE (Progressive Genome Segment Enhancement), that discovers a set of genomic segments that are predictive of MIC and compared it to two state-of-the-art approaches: (1) an annotation-based approach that uses a curated database of AMR genes to build model inputs; and (2) a *k*-mer-based approach that does not rely on prior knowledge of AMR genetic determinants. We assessed the final set of segments in terms of their ability to identify potential molecular mechanisms of resistance.

## Results

### PGSE iteratively discovered a set of genome segments that predict MIC

**Fig. 1:**
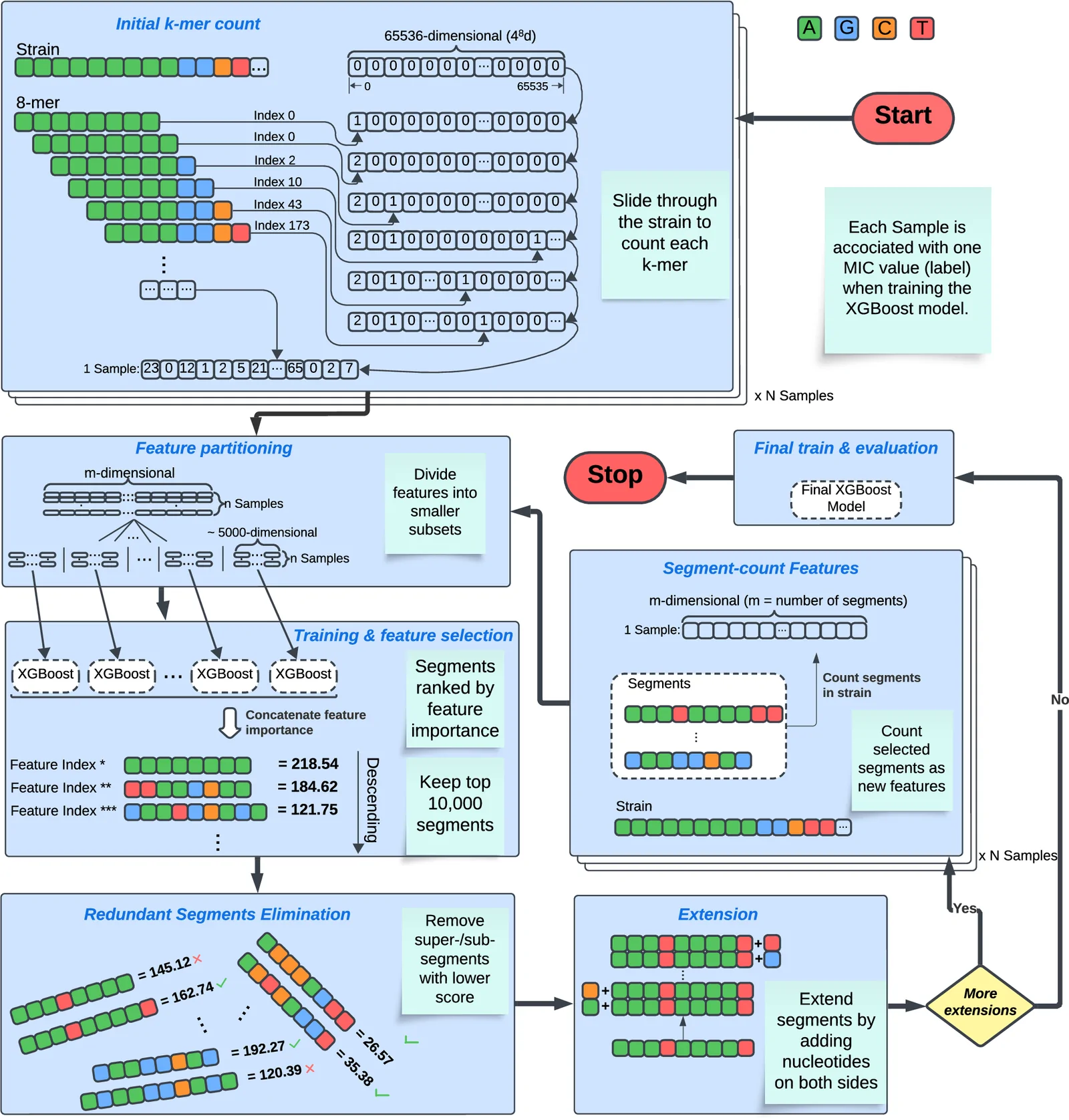
High-level overview of the PGSE algorithm. In this example, the following hyper-paramters are used: initial *k*-mer length = 8; *p* = 2.

A high-level illustration of the PGSE algorithm is shown in Fig. 1. The algorithm begins by creating an initial set of features (i.e. predictor variables) by counting overlapping *k*-mers from assembled genomes with *k* = 6. In each iteration, this feature set is divided into smaller subsets to manage computational demand, each containing approximately 5000 features. A separate gradient-boosted decision tree (XGBoost) model was trained on each subset to predict MIC. Feature importance scores, which indicate the contribution of each feature to the model’s predictive performance, were then extracted from each model.

The algorithm combined these importance scores across subsets and selected the top 10,000 features for further refinement. Next, the algorithm removed redundant segments — if one segment completely appeared within another longer segment, the algorithm kept only the segment with the higher importance score. The remaining sequences were then extended by adding all possible nucleotide combinations to both ends, creating longer segments up to a predefined extension length (set to 2 nucleotides in this study). Sequences that had already been extended in prior rounds were not re-extended to ensure efficiency and promote convergence. No segments were removed in the first iteration, as all segments had the same length of 6 nucleotides.

Subsequently, the algorithm counted occurrences of these retained and extended segments across all genomes. This updated set of counts became the new feature space for the next iteration. The process repeated until no further extensions improved the predictive performance, i.e. all newly extended segments had lower importance than the original one, at which point the algorithm converged. The final output included a trained XGBoost model and a compact set of variable-length genomic segments that were predictive of the MIC.

An open-source implementation of the PGSE algorithm is available for Python and R at: https://github.com/yinzheng-zhong/PGSE. An online version is also available at: https://molecularmic.com.

### Challenge isolates had diverse antimicrobial resistance phenotypes

The clinical strains used in this study were predominantly blood (515/762, 68%) and urinary isolates (158/762, 21%), obtained from a single laboratory serving a majority of the city of Liverpool, UK, from 2017 to 2021. After excluding MICs that did not meet predefined quality control criteria, 6080 MICs for 10 drugs were available. The proportion of resistant strains varied from 0.37% to 87.87% (tigecycline and amoxicillin, respectively) as defined using EUCAST breakpoints^13^.

**Fig. 2:**
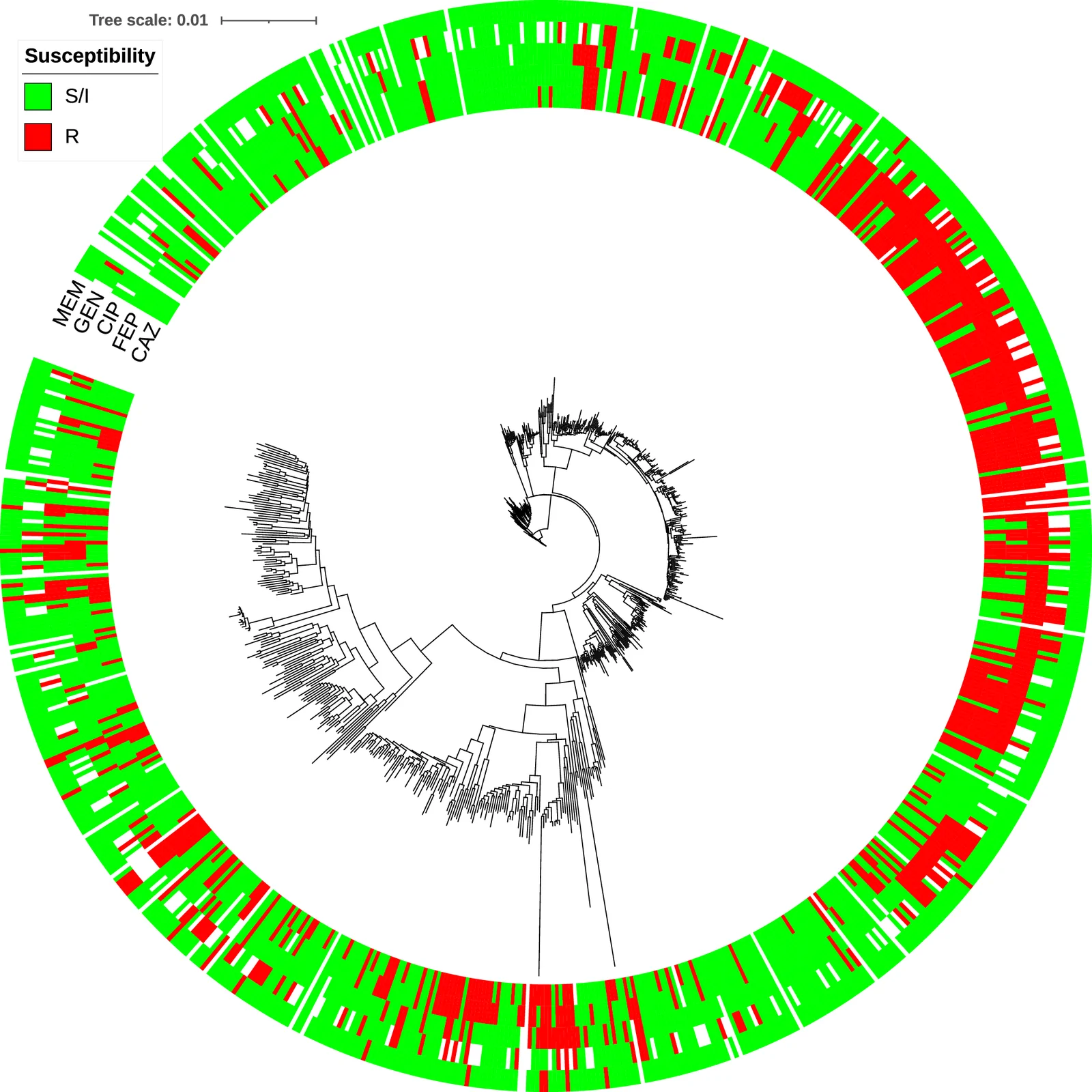
Phylogenetic tree of the *E. coli* strains used in this study. The outer rings show strain susceptibility to ciprofloxacin (CIP), ceftazidime (CAZ), cefepime (FEP), gentamicin (GEN) and meropenem (MEM).

Despite the broad range of phenotypes in the study strains, some antimicrobials had imbalanced distributions of MICs. For example, amoxicillin and amoxicillin-clavulanic acid were predominantly resistant, while amikacin, chloramphenicol, and tigecycline were predominantly susceptible (see Table 1). Imbalanced data leads to machine learning models that are trained almost exclusively on the majority phenotype, which can result in poor generalisation to minority phenotypes^14^. Therefore, subsequent machine learning analyses were focused on antimicrobials with a broad range of MICs (ceftazidime, ciprofloxacin, cefepime, gentamicin, and meropenem); the results for the remaining antimicrobials are available in the supplementary material. After annotating genomes against known AMR genes, 187 unique AMR genes were identified. The most commonly detected AMR genes were *gyrA* S83L (*n* = 339), conferring resistance to quinolones, *bla*_TEM-1_ (*n* = 314), conferring resistance to penicillins, *parC* S80I (*n* = 265), conferring resistance to quinolones, *gyrA* D87N (*n* = 254), conferring resistance to quinolones, *parE* I529L (*n* = 237), conferring resistance to quinolones. A phylogenetic tree of the study strains is shown in Fig. 2.

**Table 1:** Number of isolates by MIC value

Antimicrobial	*n*	<0.03	0.03	0.06	<0.125	0.125	0.25	0.5	1	2	4	>4	8	16	>16	32	64	>64
Amoxicillin/clavulanic acid	450	0	0	0	0	0	0	0	0	4	30	0	25	3	0	1	6	381
Amikacin	717	3	0	0	0	0	3	7	43	338	210	0	75	30	0	4	0	4
Amoxicillin	544	0	0	0	0	0	0	0	0	2	39	0	25	1	0	0	12	465
Ceftazidime	721	2	2	52	0	153	129	67	35	45	24	0	33	42	0	48	41	48
Chloramphenicol	709	7	0	3	0	0	3	7	72	320	206	0	38	5	0	7	10	31
Ciprofloxacin	703	321	11	28	0	40	30	7	4	2	4	256	0	0	0	0	0	0
Cefepime	722	186	0	96	0	90	50	33	18	21	23	0	35	31	0	28	30	81
Gentamicin	532	0	0	0	1	0	9	25	138	182	26	0	11	4	0	3	17	116
Meropenem	711	50	184	283	0	28	33	74	30	5	7	0	3	5	9	0	0	0
Tigecycline	271	0	4	77	49	77	61	2	1	0	0	0	0	0	0	0	0	0

### Genome segments captured known and novel resistance determinants

**Fig. 3:**
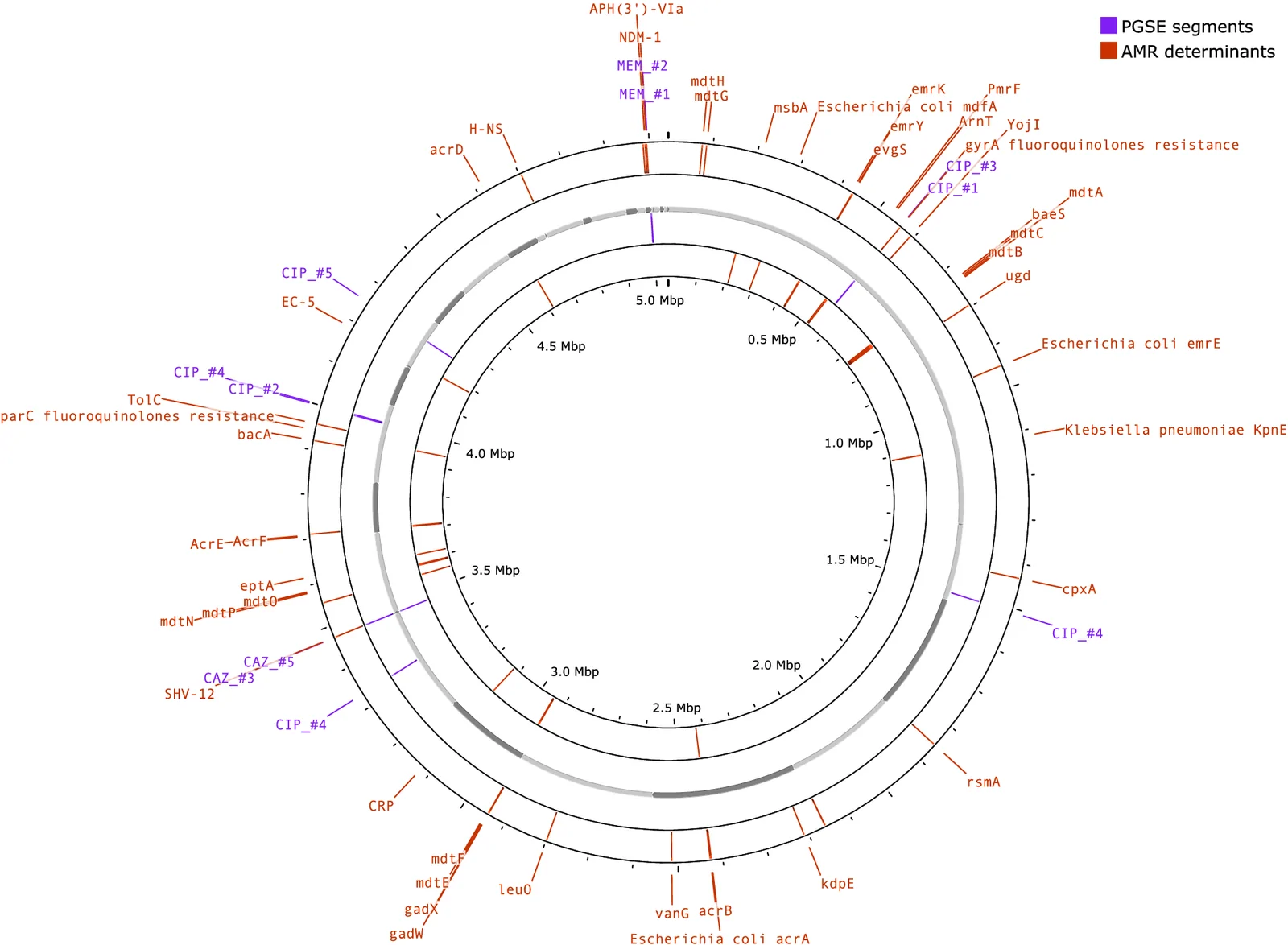
Example genome annotated with antimicrobial resistance determinants and PGSE segments. Annotations are ordered by decreasing importance; CAZ = ceftazidime, CIP = ciprofloxacin, MEM = meropenem. The strain reference number is H8.

After training, each PGSE model discovered a set of segments—i.e. variable-length nucleotide sequences—that were used to predict the MIC. To understand how these segments were used by PGSE to predict MIC, we performed a *post hoc* analysis of their relationship with functional elements in the genome. Segments were ranked by feature importance (estimated from the XGBoost gain metric), and the top 5 segments were analysed. A subset of strains containing the top segments was selected and annotated for gene coding sequences, including resistance determinants; PGSE segments were added as additional features. An example strain is shown in Fig. 3. For each PGSE segment, the closest annotated genes were recorded, and we explored the mechanistic relationship between the segments and known AMR pathways — the results are shown in Table 2. A plausible resistance determinant, or an indirect relationship to one, was found for almost all analysed segments.

**Table 2:** Genomic analysis of each PGSE model’s five segments, ranked by feature importance

Rank	Sequence	Prevalence *n* (%)	Feature importance (%)	Length (bp)	Closest gene	Distance to closest gene (bp)
Ceftazidime						
1	CCTAAATTCCACG	245 (32.2%)	10	13	*bla*_CMY-42_	100
					*bla*_CTX-M-15_	50
					*bla*_CTX-M-14_	40
2	CAATGATACTTAATAA	222 (29.1%)	5	16	*bla*_CMY-42_	250
					*bla*_CTX-M-15_	90
					*bla*_CTX-M-14_	90
3	CCAGCTGTCGGG	206 (27%)	3	12	*bla*_SHV-12_	0
					*bla*_CTX-M-15_	0
4	AAGAATTTAGAC	245 (32.2%)	2	12	*bla*_CMY-42_	150
					*bla*_CTX-M-15_	100
					*bla*_CTX-M-14_	180
5	GTGAAGGCCGATA	9 (1.2%)	2	13	*bla*_SHV-12_	20
Ciprofloxacin						
1	GCCAAGTCACCA	339 (44.5%)	21	12	*gyrA*	0
2	CGTTTAGATCCGC	459 (60.2%)	3	13	*parC*	32 K
3	ACCGCCAAGTCA	347 (45.5%)	3	12	*gyrA*	0
4	TCTGCAATTACA	752 (98.7%)	3	12	*parC*	34 K
5	AGTTCCTCGTCTG	432 (56.7%)	2	13	*paeA*	0
Gentamicin						
1	CCGATGCTTGGAA	174 (22.8%)	34	13	*aac(3)-Iid*	0
2	AGCTCGGTCGCC	175 (23%)	6	12	*aac(3)-Iid*	0
3	TATCTTGAGAAGCA	173 (22.7%)	3	14	*aac(3)-Iid*	0
4	CCTACCTAGGA	26 (3.4%)	3	11	N/A	
5	GATCGCCATGGGCA	3 (0.4%)	2	14	*rmtB*	860
Meropenem						
1	CCCGCGGCCGGCGC	148 (19.4%)	12	14	*trbA*	0
					*dsbD*	0
2	CGTGCAGCTGCAGC	15 (2%)	6	14	*trpF*	0
3	ATCCGCGGAGTA	182 (23.9%)	2	12	*aac(3)-Iid*	890
4	GGTTTTAGTGATA	20 (2.6%)	2	13	Glycosyltransferases (multiple)	0
5	CAGTCTCCGAGATG	15 (2%)	2	14	Transcription regulators (multiple)	0

The closest relevant gene/s and approximate distance are shown. Since PGSE does not differentiate between a segment and its reverse complement, only the canonical sequence is shown (i.e., the lexicographically smallest between the segment and its reverse complement). The prevalence column shows the number and percentage of strains in which the segment was detected. Results for the antimicrobials that were excluded from the primary analysis due to imbalanced data are available in the supplementary material.

The gentamicin model segments were located on, or adjacent to, the known aminoglycoside resistance determinants *aac(3)-IId*, which encodes an aminoglycoside-modifying enzyme, and *rmtB*, a 16S rRNA methylase gene that confers high-level aminoglycoside resistance^15,16^. For ciprofloxacin, the top 4 segments were associated with point mutations in *gyrA* and *parC*, reflecting the similar results seen in the annotation model. The 5th segment was found to be present in the gene *paeA* (formerly named *yrfL*). Previous research has associated this gene with DNA damage response in silico, and an *E. coli paeA* deletion mutant was experimentally shown to be more sensitive to nalidixic acid, a fluoroquinolone antimicrobial, suggesting the mechanistic role for the identified by PGSE^17^.

**Fig. 4:**
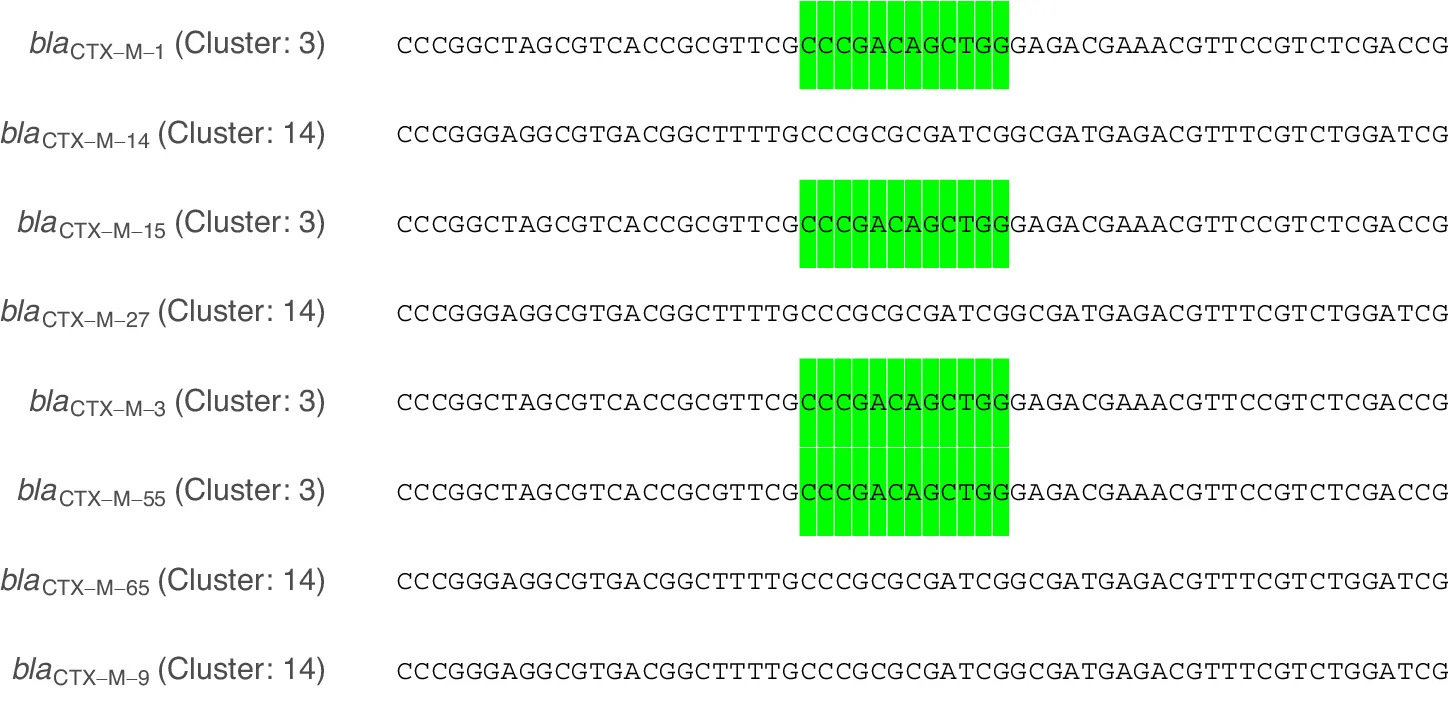
Section of aligned *bla*_CTX-M_ genes from study isolates, with the PGSE ceftazidime model segment 3 highlighted in green (CCCGACAGCTGG). The presence or absence of the segment differentiates between genes in the *bla*_CTX-M-14_ phylogenetic cluster from the *bla*_CTX-M-3_ cluster.

The ceftazidime segments reflected a heterogenous mixture of ß-lactamases that confer resistance to extended-spectrum cephalosporins. Unlike the gentamicin and ciprofloxacin models, most of the ceftazidime segments were not specific to a single resistance determinant. For example, while segments 1–4 were associated with *bla*_CTX-M-15_, which is a common extended-spectrum beta-lactamase, they were also found in or adjacent to other ß-lactamase genes such as *bla*_CMY-42_ AmpC (segments 1,2, and 4) and *bla*_SHV-12_ (segment 3). With the exception of one isolate that had *bla*_CTX-M-8_, all *bla*_CTX-M_ variants in the dataset belonged to the *bla*_CTX-M-3_ (which includes *bla*_CTX-M-15_) and *bla*_CTX-M-14_ phylogenetic clusters^18^. Segment 3 was noted to be present in *bla*_CTX-M_ genes within the *bla*_CTX-M-3_ cluster, but not in the *bla*_CTX-M-14_ cluster (Fig. 4), suggesting that PGSE segments captured phylogenetic relationships between resistance determinants.

In contrast, the meropenem model segments appeared to capture phylogenetically unrelated mechanisms of meropenem resistance. In the study strains, *bla*_OXA-48_ and *bla*_NDM_ were the most common carbapenemases (*n* = 134 and *n* = 15, respectively). Segment 1, which had the highest feature importance, matched to both *TrbA*, a conjugal transfer protein found on *bla*_OXA-48_ plasmids, and *DsbD*, a protein-disulfide reductase that is part of the conserved *ble*–*trpF*–*dsbD* plasmid region that flanks *bla*_NDM_^19,20^. This segment had a sensitivity of 99% and specificity of 96% for the detection of *bla*_OXA-48_ or *bla*_NDM_ in this dataset. The second segment matched *trpF*, also within the conserved region adjacent to *bla*_NDM_. Together, the top two segments captured the presence or absence of the two most common carbapenemases in the dataset. The remaining meropenem segments (3–5) captured co-resistance to other antimicrobials (segment 3 matched to the aminoglycoside-resistance gene *aac(3)-IId*), or to non-specific glycosyltransferases and transcription regulators (segments 4 and 5, respectively).

### PGSE predicted MIC with low memory requirements and without a priori knowledge of resistance determinants

**Fig. 5:**
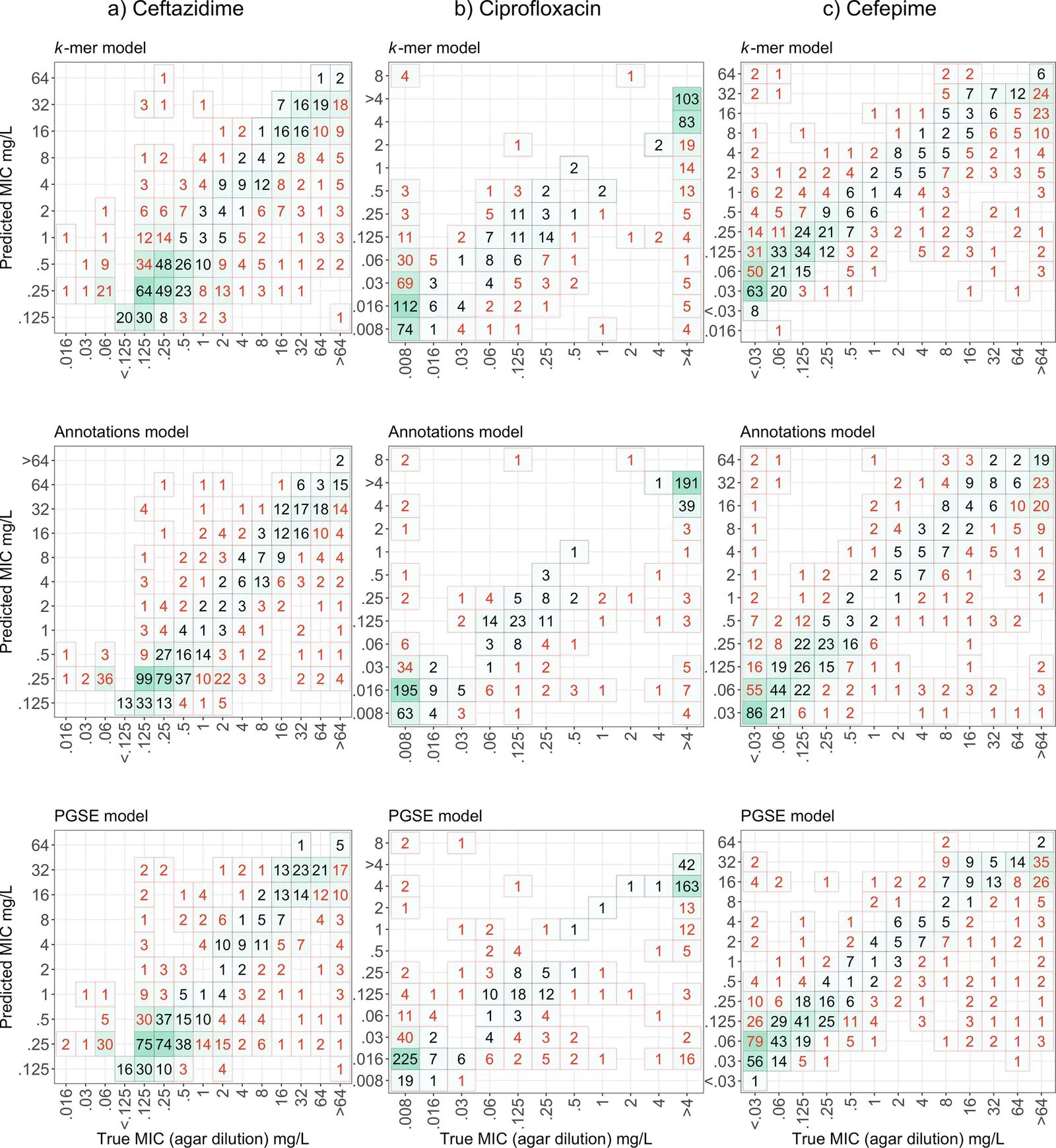
Predicted MICs from ceftazidime, ciprofloxacin, and cefepime models evaluated against true MIC, measured using agar dilution. The first row of panels shows results for *k*-mer sub-models, second row annotations sub-models, and third row PGSE sub-models. The first column of panels shows results for ceftazidime (**a**), the second column for ciprofloxacin (**b**), and the third column for cefepime (**c**).

**Fig. 6:**
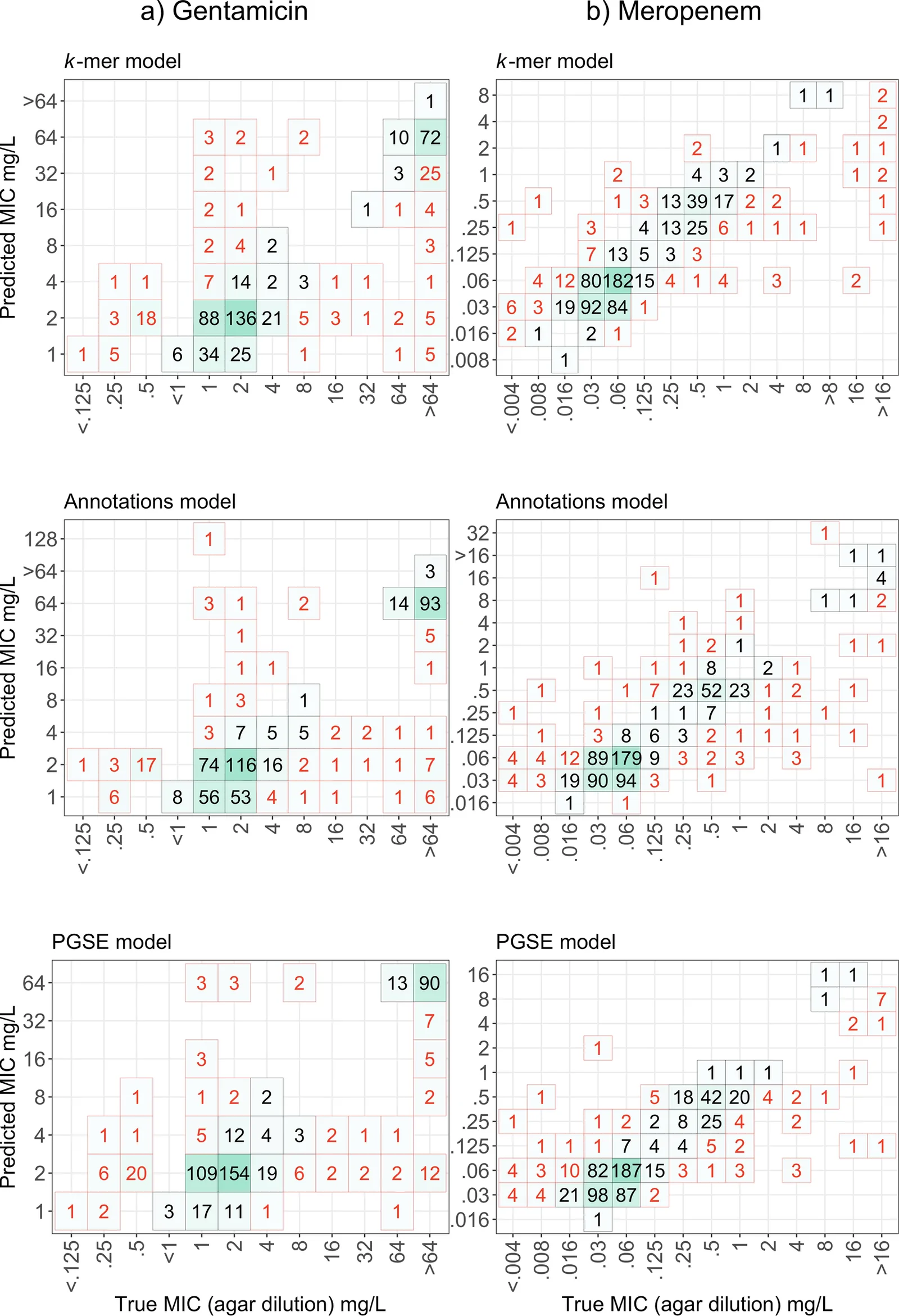
Predicted MICs from gentamicin and meropenem models evaluated against true MIC, measured using agar dilution. The first row of panels shows results for *k*-mer sub-models, second row annotations sub-models, and third row PGSE models. The first column of panels shows results for gentamicin (**a**) and the second column for meropenem (**b**).

Each model’s predicted MICs were evaluated against observations obtained from agar dilution using essential (EA) and categorical agreement (CA) — the results are summarised in Table 3. Figures 5 and 6 show the predicted vs observed MICs for each antimicrobial.

**Table 3:** Essential and categorical agreement rates for *k*-mer (*k* = 10), annotation (Annot.) and PGSE (starting *k* = 6) models

Antimicrobial	*n* MICs	Resistant *n* (%)	Model	EA *n* (%)^a^	mE *n* (%)^b^	ME *n* (%)^c^	vME *n* (%)^d^	*r*^e^
Ceftazidime	721	212 (29.4)	*k*-mer	415 (57.6)	127 (17.6)	13 (1.8)	26 (3.6)	0.77
			Annot.	491 (68.1)	104 (14.4)	15 (2.1)	20 (2.8)	0.80
			PGSE	453 (62.8)	96 (13.3)	16 (2.2)	26 (3.6)	0.79
Ciprofloxacin	703	266 (37.8)	*k*-mer	460 (65.4)	31 (4.4)	5 (0.7)	29 (4.1)	0.86
			Annot.	588 (83.6)	12 (1.7)	6 (0.9)	29 (4.1)	0.89
			PGSE	531 (75.5)	19 (2.7)	11 (1.6)	27 (3.8)	0.87
Cefepime	722	205 (28.4)	*k*-mer	374 (51.8)	78 (10.8)	9 (1.2)	33 (4.6)	0.78
			Annot.	409 (56.6)	61 (8.4)	13 (1.8)	29 (4)	0.80
			PGSE	380 (52.6)	57 (7.9)	12 (1.7)	32 (4.4)	0.80
Gentamicin	532	177 (33.3)	*k*-mer	418 (78.6)	0 (0)	39 (7.3)	44 (8.3)	0.81
			Annot.	451 (84.8)	0 (0)	21 (3.9)	41 (7.7)	0.82
			PGSE	437 (82.1)	0 (0)	32 (6)	45 (8.5)	0.82
Meropenem	711	14 (2)	*k*-mer	620 (87.2)	14 (2)	0 (0)	9 (1.3)	0.78
			Annot.	624 (87.8)	15 (2.1)	1 (0.1)	5 (0.7)	0.77
			PGSE	627 (88.2)	19 (2.7)	0 (0)	3 (0.4)	0.80

Predictions were considered to be in essential agreement if the predicted MIC was within one doubling dilution of the reference MIC. For categorical agreement, minor error (me) was defined as *I* on one method and *S/R* on the other method; major error (ME) was defined as a prediction of *R* and *S* on the reference method; and very major error (vME) was defined as a prediction of *S* and *R* on the reference method.

^a^Essential agreement (EA).

^b^Minor error (mE).

^c^Major error (ME).

^d^Very major error (vME).

^e^Pearson correlation coefficient (*r*).

For the first modelling approach, models were trained to predict MIC using input features generated from annotated genome data^21^. These models had the highest rate of essential agreement (±1 dilution) with agar dilution MIC (median EA 75.5%, ranging from 52.6% for cefepime to 88.2% for meropenem). When all antimicrobials were pooled, the essential agreement rate was 75.6%. To understand the extent to which a priori knowledge of AMR genes contributed to the predictive performance of the annotation models, we extracted feature importance from the XGBoost models — the top three genes for each antimicrobial sub-model are shown in Table 4. These top three genes were responsible for between 55.7% and 95.6% of the total feature importance. Resistance determinants with high feature importance were generally of the same or related antimicrobial class as the antimicrobial being modelled.

**Table 4:** Top three genes with highest feature importance for each annotation model

Gene symbol	Feature importance (%)	Cumulative feature importance (%)	Resistance activity
Ceftazidime			
*bla*_CTX-M-15_	47.6	47.6	Cephalosporin
*bla*_CTX-M-27_	4.3	51.9	Cephalosporin
*bla*_SHV-12_	4.2	56.1	Cephalosporin
Ciprofloxacin			
*parC* S80I	58.5	58.5	Quinolone
*gyrA* D87N	31.0	89.5	Quinolone
*gyrA* S83L	6.1	95.6	Quinolone
Cefepime			
*bla*_CTX-M-15_	48.7	48.7	Cephalosporin
*bla*_CTX-M-27_	4.1	52.8	Cephalosporin
*bla*_CTX-M-14_	2.9	55.7	Cephalosporin
Gentamicin			
*aac(3)-IId*	43.7	43.7	Gentamicin
*aac(3)-IIe*	36.6	80.2	Gentamicin
*ant(2”)-Ia*	2.1	82.3	Gentamicin, kanamycin, tobramycin
Meropenem			
*bla*_OXA-48_	34.3	34.3	Carbapenem
*ble*	26.8	61.1	Bleomycin
*bla*_KPC-2_	4.9	66.0	Carbapenem

The ble gene confers resistance to the cancer chemotherapeutic agent bleomycin and is found systematically downstream of *bla*_NDM_. The relatively high feature importance of ble on meropenem MIC is likely due to colinearity with *bla*_NDM_.

**Fig. 7:**
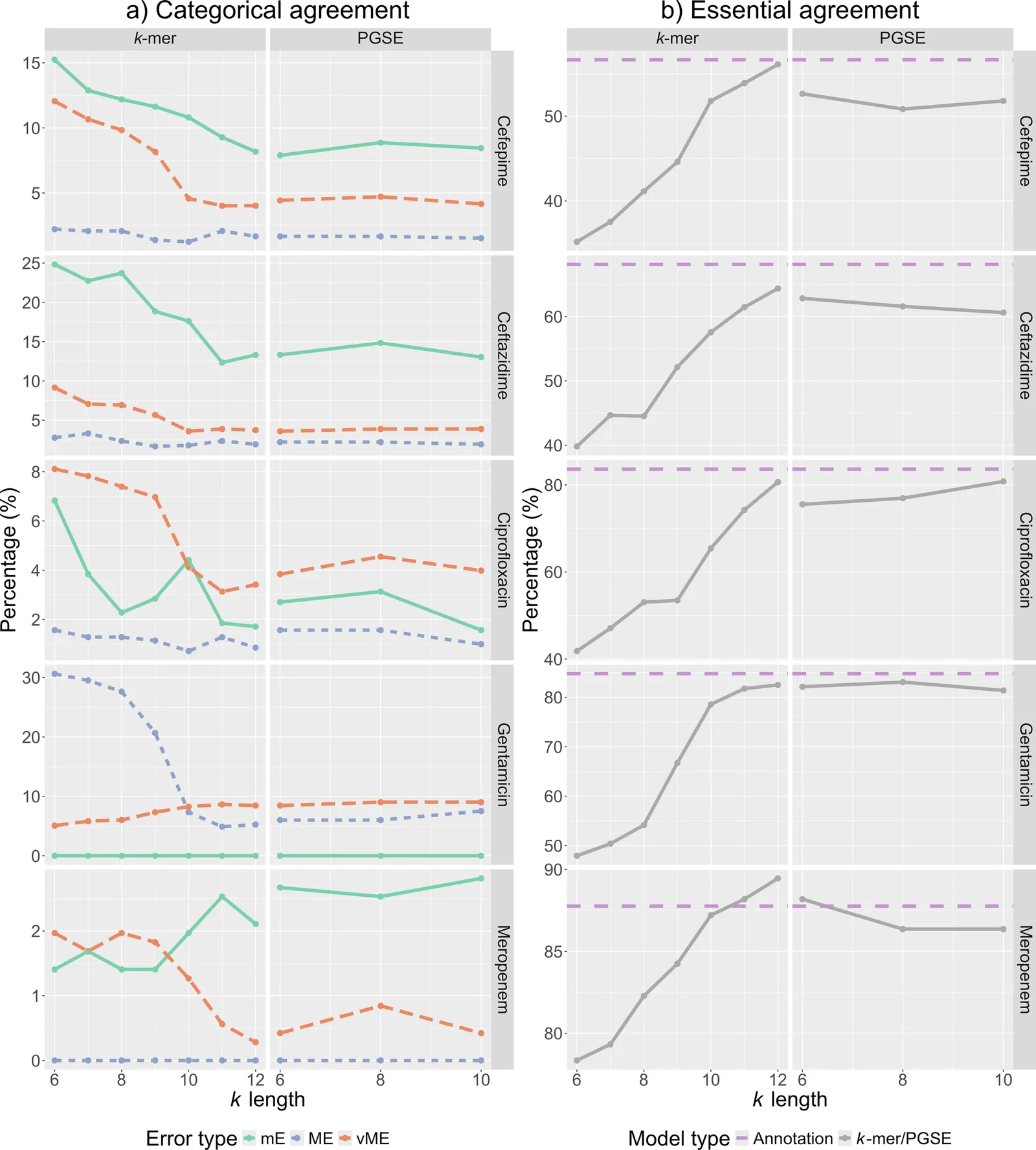
Essential agreement and error rates for the *k*-mer models across different lengths of *k*, compared to PGSE with different starting values for *k*. **a** shows results using categorical agreement as an evaluation metric, while **b** shows results using essential agreement; mE = minor error (I on one method S or R on the other method); ME = major error (prediction of R; S on the reference method); and vME = very major error (prediction of S; R on the reference method). Essential agreement rate for annotation models (right) was a fixed value (horizontal dashed line).

For the next modelling approach, models were trained to predict MIC from *k*-mer counts of fixed length. Models trained using *k*-mer counts had lower rates of EA and CA compared to the annotation models (median EA 65.4%, ranging from 51.8% for cefepime to 87.2% for meropenem; 67.5% when pooled across all antimicrobials), but their predictions did not require a priori information on resistance determinants. The performance of *k*-mer models varied with respect to the *k* value — EA and CA rates broadly improved with larger *k* values, but with an increased computational cost due to the increased feature dimensionality (Fig. 7). A value of 10 was selected, as it was used in previous research and balanced predictive performance against computational cost^8^.

For the final modelling approach, models were trained using our novel PGSE algorithm. We then compared the predictive performance of PGSE to the *k*-mer and annotation models. The PGSE models had EA and CA rates comparable to the annotation models, and better than the *k*-mer models (median 75.5%, ranging from 52.6% for cefepime to 88.2% for meropenem; 71.6% when pooled across all antimicrobials). Like the *k*-mer models, the PGSE models did not require a priori information on resistance determinants. However, in contrast to the *k*-mer models, the sensitivity of the PGSE models to the initial *k*-mer length hyper-parameter was minimal (see Fig. 7) — a value of 6 was selected. The models trained using PGSE required significantly less computational resources than the *k*-mer models, assessed using an 80–20% split of the ceftazidime dataset. When using an initial *k* value of 6, the PGSE model used a peak of 23.6GB of RAM (mean 19.5GB). The computational time required for the entire PGSE process was 36 min. In contrast, a *k*-mer model (using *k* = 10) required 71 min to fit (excluding hyper-parameter tuning) using a maximum 38.8GB of RAM (mean 30.1GB). Models with *k* > 10 were unable to be run on a machine with 64GB of RAM, due to insufficient available memory.

### PGSE models had higher generalisability when using external data

To assess model generalisability — defined as the performance of a model on unseen data — an external dataset of *E. coli* WGS data was retrieved from the BV-BRC database^22^; 4755 genomes were available. Each machine learning model was applied to the external dataset, and the predicted MIC was compared to the metadata provided by the BV-BRC database. Although we explored comparing MIC values, we found that this was unreliable due to systematic differences in MIC methodology, particularly differences in censoring at the extreme ranges. Instead, we used binary measures of predictive performance — the F1 score, sensitivity, and specificity — for external validation. The F1 score obtained on the external dataset was compared to the internal dataset — the results are shown in Table 5. The PGSE models had the highest F1 score on external data (median 0.83 [range 0.56–0.9]; 0.85 when pooled across all antimicrobials), followed by the annotation models (median 0.75 [range 0.56–0.9]; 0.82 when pooled across all antimicrobials), and the *k*-mer models (median 0.74 [range 0.32–0.78]; 0.74 when pooled across all antimicrobials). The PGSE models’ F1 score was very similar on the internal and external datasets, with the exception of meropenem.

**Table 5:** External model validation: summary of F1 scores, sensitivity, and specificity from the internal and external (BV-BRC) datasets for the *k*-mer, annotations, and PGSE models

	Ceftazidime (*n* = 4260; 43.2% susceptible)								
Model	Annotation			*k*-mer			PGSE		
Dataset	Internal	External (BV-BRC)	Gen. gap	Internal	External (BV-BRC)	Gen. gap	Internal	External (BV-BRC)	Gen. gap
F1 score	0.83	0.81	−0.02	0.79	0.78	−0.01	0.83	0.83	0.00
Sensitivity	0.78	0.72	−0.06	0.77	0.71	−0.06	0.78	0.81	0.03
Specificity	0.93	0.93	0.00	0.90	0.85	−0.05	0.93	0.83	−0.10
	Ciprofloxacin (*n* = 4501; 40.7% susceptible)								
Model	Annotation			*k*-mer			PGSE		
Dataset	Internal	External (BV-BRC)	Gen. gap	Internal	External (BV-BRC)	Gen. gap	Internal	External (BV-BRC)	Gen. gap
F1 score	0.91	0.90	−0.01	0.91	0.75	−0.16	0.91	0.90	−0.01
Sensitivity	0.87	0.82	−0.05	0.88	0.60	−0.28	0.88	0.82	−0.06
Specificity	0.98	1.00	0.02	0.97	0.98	0.01	0.96	0.99	0.03
	Cefepime (*n* = 2877; 42.9% susceptible)								
Model	Annotation			*k*-mer			PGSE		
Dataset	Internal	External (BV-BRC)	Gen. gap	Internal	External (BV-BRC)	Gen. gap	Internal	External (BV-BRC)	Gen. gap
F1 score	0.87	0.75	−0.12	0.83	0.66	−0.17	0.85	0.84	−0.01
Sensitivity	0.84	0.62	−0.22	0.80	0.51	−0.29	0.81	0.78	−0.03
Specificity	0.95	0.97	0.02	0.94	0.94	0.00	0.95	0.90	−0.05
	Gentamicin (*n* = 4643; 77.4% susceptible)								
Model	Annotation			*k*-mer			PGSE		
Dataset	Internal	External (BV-BRC)	Gen. gap	Internal	External (BV-BRC)	Gen. gap	Internal	External (BV-BRC)	Gen. gap
F1 score	0.81	0.75	−0.06	0.76	0.74	−0.02	0.77	0.79	0.02
Sensitivity	0.77	0.74	−0.03	0.75	0.74	−0.01	0.75	0.70	−0.05
Specificity	0.94	0.93	−0.01	0.89	0.93	0.04	0.91	0.98	0.07
	Meropenem (*n* = 4207; 97.3% susceptible)								
Model	Annotation			*k*-mer			PGSE		
Dataset	Internal	External (BV-BRC)	Gen. gap	Internal	External (BV-BRC)	Gen. gap	Internal	External (BV-BRC)	Gen. gap
F1 score	0.56	0.56	0.00	0.40	0.32	−0.08	0.70	0.56	−0.14
Sensitivity	0.46	0.42	−0.04	0.25	0.19	−0.06	0.54	0.39	−0.15
Specificity	0.99	1.00	0.01	1.00	1.00	0.00	1.00	1.00	0.00

The generalisation gap (Gen. gap) is calculated as the difference between the external and internal F1 score/sensitivity/specificity — values < 0 indicate that perfomance on external data is worse than internal; values > 0 indicate better performance on external data.

On the external dataset, PGSE’s absolute F1 score on meropenem (0.56) was higher than that of the *k*-mer model (0.32), and equal to that of the annotation model. However, this was lower than the F1 score on the internal dataset (0.7). This difference in performance was likely due to differences in the distribution of carbapenem resistance mechanisms between the datasets (see Table S4 for a full breakdown). In particular, *bla*_OXA-48_ was far more common in the internal dataset (18.4% of strains) compared with the external dataset (0.04% of strains). This particular carbapenem-resistant phenotype generally has meropenem MIC values that are classed as susceptible on clinical breakpoints (the median meropenem MIC of strains with *bla*_OXA-48_ was 0.5 mg/L in the internal dataset), which likely explains the discrepancy in F1 scores.

## Discussion

In this work, we report a novel algorithm, PGSE, that uses WGS data to discover a set of genomic segments that can be used to predict MIC. We compared and evaluated PGSE against two state-of-the-art modelling approaches that use *k*-mer counts or annotated AMR genetic determinants as inputs^3,8,9,23–25^. The PGSE algorithm has multiple important advantages over these approaches. Firstly, it is computationally efficient, requiring significantly less memory and time to train compared to *k*-mer models, enabling the use of larger datasets. It was possible to train PGSE models on our study dataset using workstation-level hardware with 64GB of RAM. These computational improvements do not come at the expense of predictive performance — the PGSE models had better essential and categorical agreement compared to *k*-mer models with *k* = 10.

Additionally, PGSE is less sensitive to the choice of *k* length, compared to *k*-mer models (Fig. 7). While larger *k* values preserve data granularity and capture more information, they also increase input dimensionality and lead to sparser *k*-mer counts, potentially leading to overfitting^26^. In practice, *k* is usually chosen pragmatically, depending on available computational resources. The *k* hyper-parameter in PGSE governs the length of the initial *k*-mer counts that are later extended — a small value (*k* = 6) gave results comparable to traditional *k*-mer models with *k* > 10 (which are usually too computationally expensive to train for large datasets).

Finally, PGSE identifies variable-length genome segments that are predictive of MIC — in effect, discovering a data-driven collection of AMR genetic determinants without requiring an explicit mechanistic understanding of the resistance mechanisms. Unlike traditional annotation-based models, PGSE does not require a database of known AMR genetic determinants; therefore, it is not limited by the comprehensiveness of such a database. These segments may be used for downstream applications beyond predicting MIC, such as identifying novel resistance mechanisms or designing assays against novel molecular targets. Some segments were found to have a direct relationship with known resistance determinants. Others indirectly matched conserved genomic regions that are plausibly associated with reduced antimicrobial susceptibility. While some segments appeared to have a phylogenetic rationale (e.g. ceftazidime segment 3, which was only found in *bla*_CTX-M-3_ cluster genes), others captured information about multiple, but phylogenetically unrelated, resistance mechanisms (e.g. meropenem segment 1, which matched to both *bla*_OXA-48_ and *bla*_NDM_ carbapenemases). We speculate that the latter may be due to the PGSE algorithm’s approach to segment extension, which terminates when an extension does not improve the model’s predictive performance. Thus, PGSE keeps each segment’s length optimal, capturing multiple resistance motifs. While we considered a similar analysis on the *k*-mer models’ features, we found that this was less practical due to the low specificity of the *k*-mers generated for this study (i.e. many *k*-mers were present multiple times in an individual genome).

We used a bespoke strain bank to ensure that a broad range of MIC phenotypes was available for model training. In contrast, most similar research works have used existing datasets of strains that were collected for other purposes, such as for surveillance^8,23,24^. Uncommon but clinically important resistance phenotypes may be under-represented in such datasets. Models trained using imbalanced data can be misleading despite apparently good essential and categorical agreement. Furthermore, we trained and validated models first on the internal dataset, and then tested them further on an external dataset generated by other researchers. This gives a realistic assessment of the models’ generalisability — a key consideration in machine learning^27^, since performance may be different on datasets with different distributions of resistance mechanisms, or where AST was measured using different laboratory methods. Related research has generally used a single dataset, or multiple datasets were combined into a single dataset, which may not provide a realistic assessment of model generalisability^3,8,9,24,25^. The models trained using PGSE generalised well to external data. We attribute this to PGSE’s data-driven design that does not rely on known resistance determinants, and the heterogeneous training dataset that was used for this study.

As MIC prediction from WGS matures, new opportunities are presented by PGSE. Although the MIC phenotype remains the gold standard method of AST, it has important limitations that may be partially overcome through the use of genomic data. Since phenotypic MIC measurements are affected by intra- and inter-laboratory variation, results may not be reproducible from day-to-day and between laboratories^28^. Unlike phenotypic MICs, the prediction of MIC from genomic data using a machine learning model can be considered to be a fixed value. Therefore, MIC distributions can be reliably compared across time points, laboratories, or regions, which is important for AMR surveillance. Furthermore, almost all methods for measuring MIC (e.g. broth microdilution and gradient strips) are inherently imprecise due to the censoring of MIC values. For example, an MIC result of 64 mg/L is technically an MIC within the range of 32–64 mg/L. This imprecision may have implications when quantifying pharmacodynamic target attainment. The outputs of the models described were continuous values that were converted to categorical values to enable essential and categorical agreement calculations. Future research can explore whether continuous MIC prediction values can reflect true differences in MIC —e.g. can the model differentiate 32.1 mg/L from 63.9 mg/L?—and whether this is clinically useful.

Another potential application for predicting MIC from genomic data is in situations where the MIC cannot be measured phenotypically. For example, while there was a commitment to increased availability of testing for AMR at the 2024 UN General Assembly High-Level Meeting on AMR, only 1–2% of laboratories in sub-Saharan Africa can undertake bacteriology testing^29–31^. The estimation of MIC from WGS may help generate additional AMR surveillance data in these regions. The use of WGS also reduces the need to transport strains for MIC measurement, which may not always be possible due to biosecurity implications.

In most clinical laboratories, algorithms like PGSE are likely to be used in conjunction with phenotypic AST methods. Generally, laboratories perform a ‘one-size-fits-all’ initial susceptibility testing panel^32^; if AMR is detected, additional panels of tests are performed, which usually require a further period of incubation. Using PGSE or other machine learning approaches, the laboratory could use WGS data to generate predictions for the entire panel of antimicrobials in the model’s database. Second-line phenotypic MIC testing can then be targeted based on the information gained from PGSE. Computationally efficient models such as PGSE would also allow for a feedback loop where new data (WGS and MICs) are used to re-train the model^5^.

This study has several limitations. Firstly, only 762 strains were used, and all were from one region in the UK; a larger and geographically more diverse dataset may improve performance by better capturing relevant resistance mechanisms. Secondly, only *E. coli* isolates were included. Future work should explore how the approach generalises to other clinically relevant species. Furthermore, while we benchmarked PGSE against two established approaches to MIC prediction from WGS (*k*-mer counts and annotated AMR genes), more approaches are possible, such as the use of nonredundant long *k*-mers^33–35^ or SNPs as model input features^3^. To understand PGSE’s scalability and robustness, formal benchmarking against a wider range of approaches is required. As the availability of open source data increases, larger datasets of diverse organisms and antimicrobials should be used when benchmarking performance.

Although the prediction of MIC from WGS is in its nascent stages, it is likely to have a significant impact on the field of AST. If performance continues to improve with larger datasets and more sophisticated models, machine learning can potentially impact clinical care and AMR surveillance. The advantages provided by our novel algorithm described here (PGSE) are an important advancement in delivering a fast, memory-efficient, and mechanism-agnostic approach to predicting AST from WGS.

## Methods

**Fig. 8:**
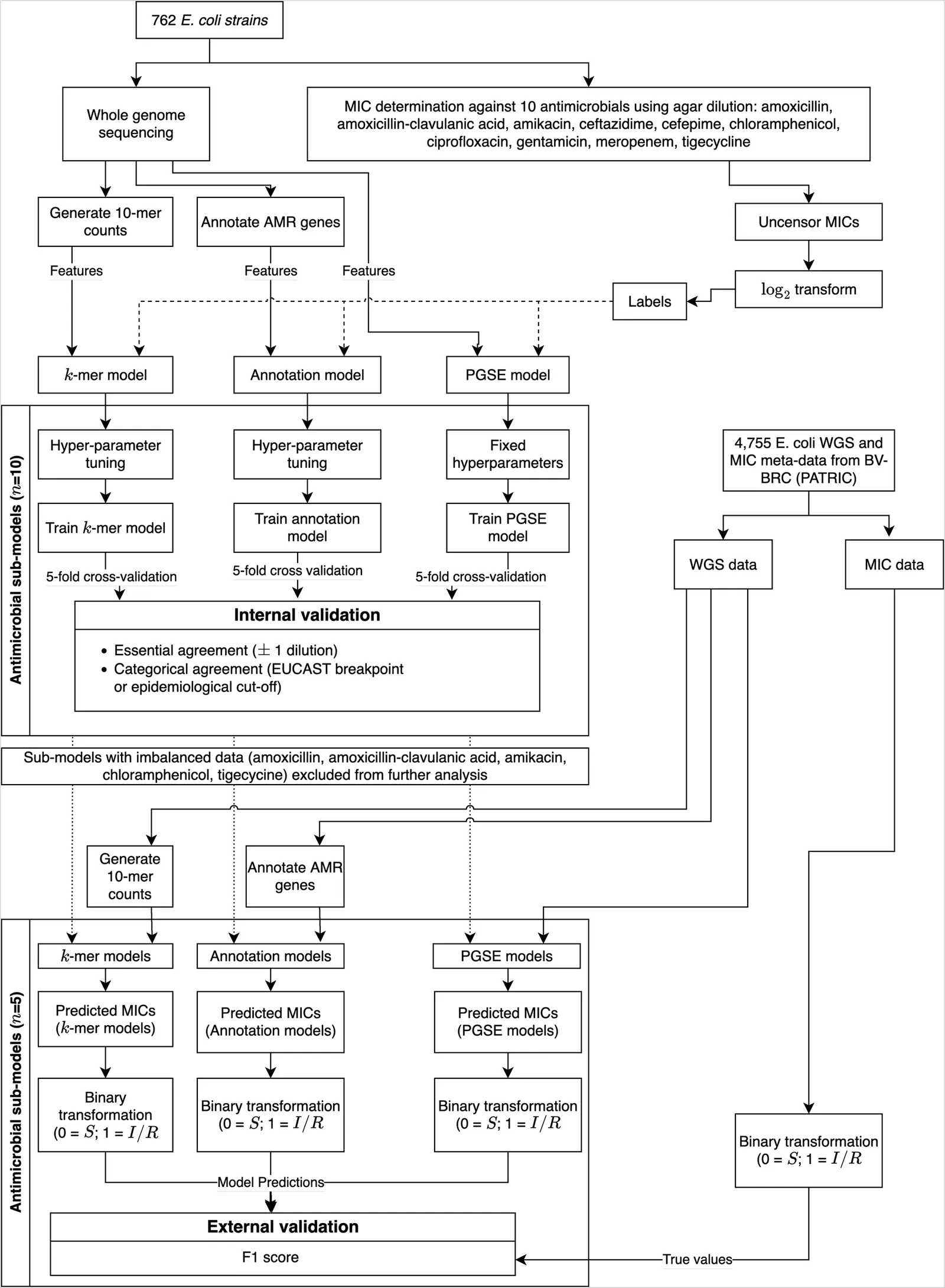
Study design flowchart. Model features generated from genomic data (*k*-mer counts or annotated genes). Model labels generated from MIC data. EUCAST = European Committee on Antimicrobial Susceptibility Testing.

A summary of the laboratory and machine learning methodology used in this study is shown in Fig. 8.

### Strains used in this study

A collection of clinical *E. coli* strains (*n* = 762) was generated by retrieving stored isolates (stored on beads at −80 °C) from Liverpool Clinical Laboratories, Liverpool, UK. To generate this collection, we first retrieved a list of all stored clinical *E. coli* isolates and their respective disk-susceptibility testing results collected between 2017 and 2021. Isolates were identified using MALDI-TOF (Bruker Daltonics, Massachusetts, USA).

Next, to generate a representative sample of Liverpool’s antibiogram, isolates were grouped into eight antimicrobial susceptibility phenotypes defined using EUCAST disk-susceptibility testing that was obtained from LCL and used for direct clinical care: (1) fully susceptible (or monoresistance to amoxicillin); (2) resistant to amoxicillin and amoxicillin-clavulanic acid; (3) quinolone and gentamicin resistant; (4) extended-spectrum beta-lactamase (ESBL) producers; (5) ESBL producers with quinolone and gentamicin resistance; (6) AmpC producers; (7) carbapenem non-susceptible and ESBL/AmpC negative; and (8) carbapenem non-susceptible and ESBL/AmpC positive. Cefpodoxime and cefoxitin susceptibility were used as a proxy for ESBL and AmpC production, respectively. Strains that were positive on molecular testing for carbapenemase genes (OXA-48, IMP, VIM, NDM, KPC) were classified as nonsusceptible to carbapenems.

Subsequently, isolates were taken equally (without replacement) from each of the eight phenotypic groups until the target number of strains was reached. If a group did not have sufficient numbers to meet the target, isolates were taken randomly (without replacement) from the rest of the groups until the target of 762 strains was reached.

### Antimicrobial susceptibility testing

Minimum inhibitory concentrations (MICs) were determined using agar dilution for the following 10 antimicrobial agents: amoxicillin, amikacin, amoxicillin-clavulanic acid, ceftazidime, cefepime, chloramphenicol, ciprofloxacin, gentamicin, meropenem, and tigecycline (MERCK, Poole UK). A previously described agar dilution protocol was used to determine the MICs^36^. Briefly, a series of doubling dilutions of each drug was dissolved in molten Mueller–Hinton agar (Fisher Scientific, Loughborough, UK) at approximately 50 °C and poured into Corning 120 cm × 120 cm square plates (Fisher Scientific). An additional plate was prepared without drug and was used as a positive (growth) control.

Study strains were retrieved from long-term −80 °C storage and sub-cultured into glycerol stocks in 96-well PCR plates. Two wells were reserved for a negative control (broth only) and the positive control strain *E. coli* ATCC 25922. On the day of testing, the glycerol stocks were sub-cultured into 96-well plates containing 100 µL of Mueller–Hinton broth (MHB). Plates were incubated at 37 °C with shaking until an OD_625_ ≥ 0.08 was reached (within 3 h). Cultures were then diluted in MHB to 0.5 McFarland standard turbidity (OD_625_ = 0.08). Cultures below 0.08 were discarded.

Cultures were inoculated onto the pre-prepared agar plates using a 96-pin replicator (Boekel Scientific, Philadelphia, USA) that delivered 1 µL of suspension to achieve approximately 10^4^ colony-forming units per spot. Plates were incubated at 37 °C for 18 h.

Plates were imaged and colony growth annotated using the AIgarMIC software with the default models as previously described^37,38^.

### Genomic pipeline and analysis

Bacterial genomic DNA was extracted using the Qiagen MagAttract HMW DNA kit (Qiagen, UK), according to the manufacturer’s instructions. Quantification of isolated DNA was performed by using the Qubit 4.0 fluorometer (Thermo Fisher Scientific, Waltham, USA). DNA quality was assessed using a Nanodrop 2000 spectrophotometer (Thermo Fisher Scientific, UK). Illumina fragment libraries were prepared using NEBNext Ultra II FS kit (E7805) on the SPT Labtech Mosquito HV platform using a 1/10 reduced volume protocol.

Final libraries were pooled in equimolar amounts using Qubit and Bioanalyzer data. The quantity and quality of each pool was assessed by Bioanalyzer and subsequently by qPCR using the Illumina Library Quantification Kit from Kapa (KK4854) on a Roche Light Cycler LC480II according to manufacturer’s instructions. Briefly, a 10 µl PCR reaction (performed in triplicate for each pooled library) was prepared on ice with 6 µl SYBR Green I Master Mix and 2 µl diluted pooled DNA (1:1000 to 1:100,000 depending on the initial concentration determined by the Qubit® dsDNA HS Assay Kit). PCR thermal cycling conditions consisted of initial denaturation at 95 °C for 5 min, 35 cycles of 95 °C for 30 s (denaturation) and 60 °C for 45 s (annealing and extension), melt curve analysis to 95 °C (continuous) and cooling at 37 °C (LightCycler® LC48011, Roche Diagnostics Ltd, Burgess Hill, UK). Following calculation of the molarity using qPCR data, template DNA was diluted to 300pM and denatured for 8 min at room temperature using freshly diluted 0.2 N sodium hydroxide (NaOH) and the reaction was subsequently terminated by the addition of 400 mM TrisCl ph = 8. To improve sequencing quality control, 1% PhiX was spiked in. The libraries were sequenced on the Illumina® NovaSeq 6000 platform (Illumina®, San Diego, USA) following the XP workflow on 1 lane of an S4 flow cell, generating 2 × 150 bp paired-end reads.

FASTQ files were trimmed to remove Illumina adapter sequences using Cutadapt version 1.2.1^39^. The option -O3 was used, ensuring the 3’ end of reads matching the adapter sequence on 3 bp or more were trimmed. The reads were further trimmed using Sickle version 1.200 with a minimum window quality score of 20. Trimmed reads shorter than 15 bp were removed, and remaining reads without a pair were discarded prior to analysis. Subsequently, all read sets were assembled using the SPAdes assembler (v3.15.4), using default parameters plus --careful and --only-assembler options^40^. To identify AMR genetic determinants, the assembled scaffolds were annotated using AMRFinderPlus version 3.11.14, using the default settings and database version 2023-07-13.2^41,42^.

Genome-wide phylogenetic analysis was performed by generating a distance matrix using Mash version 2.0^43^. The interactive tree of life (iTOL) was used to visualise the phylogenetic tree, with the genome of *E. coli* ATCC 25922 set as the tree root^44^. Visualisation of annotated genomes was conducted on the Proksee platform^45^, using the built-in implementations of Prokka (v1.14.6)^46^ and the Comprehensive Antibiotic Resistance Database (CARD) Resistance Gene Identifier (RGI) (v6.0.3)^47^.

### Data preprocessing

To generate labels for the machine learning models, the MICs generated from the agar dilution experiments were processed using the MIC and AMR R packages^48^. Firstly, MICs were uncensored by replacing values below the lowest measured concentration with the halved concentration (e.g. ≤0.5 mg/L was converted to 0.25). Next, values above highest measured concentration were replaced with the doubled concentration (e.g. >64 mg/L was converted to 128 mg/L). Finally, MICs were log-2-transformed (log_2_). Data points where the ATCC *E. coli* 25922 control strain MIC was not within the EUCAST quality control range, or without growth in the positive (growth) control plate, were excluded from further analysis.

### Model development

To train a model to predict MIC from bacterial genome data, we performed supervised machine learning using XGBoost, a gradient-boosted decision tree algorithm. We trained three model variants: (1) a *k*-mer model that used all 10-mer permutations as features; (2) an annotations model that used AMR genes from the AMRFinderPlus database as features; and (3) a model that was trained using our novel algorithm — PGSE. Each model variant consisted of 10 sub-models, one for each antimicrobial tested. Therefore, a total of 30 models were trained. The learning task was a regression problem; the model learning objective was minimisation of the regularised root mean squared error (RMSE) between the predicted and true MICs^21^. All models were supplied with the same labels — the log_2_ transformed MICs.

To generate features for the annotation model, a separate *n* by *m* matrix was generated from the gene annotations generated by AMRFinderPlus, where *n* is the number of strains and *m* is the number of unique AMR genes. Data were transformed into a binary-encoded format.

To generate features for the *k*-mer model, overlapping *k*-mer counts were generated for each bacterial strain and loaded into memory as a sparse *n* by *m* matrix, where *n* is the number of strains (762) and *m* is the number of unique canonical *k*-mers. Only the lexiconographically smallest of a *k*-mer and its reverse complement was counted (i.e. canonical *k*-mers).

During model development and evaluation, five-fold cross-validation was used. Briefly, the training data was shuffled and partitioned into *n* = 5 equal subsets, each containing a proportionately equal distribution of MIC values. Next, one fold was kept aside as a validation set, and the remaining *n*−1 folds were used as the training data for hyper-parameter tuning and model training. This process was repeated five times, so that each fold was used as a validation set once.

To estimate the optimal hyperparameters for these models, we first performed a grid search over the maximum tree depth (minimum = 4; maximum = 10) and minimum child weight (minimum = 1; maximum = 10) parameters. The optimal hyperparameters were estimated based on the validation folds, with models trained for 100 rounds using a learning rate of 0.02. The hyperparameter values that gave the lowest MSE were fixed and carried over, and the process was repeated to optimise the next group of hyperparameters: the subsample ratio (minimum = 0.5; maximum = 1.0), and the column sample by tree (minimum = 0.4; maximum = 1.0) hyperparameters.

The *k*-mer and annotation models were then trained using the tuned hyperparameters, using a learning rate of 0.005 — each XGBoost model was trained until the MSE did not improve for 10 consecutive rounds. To identify an optimal *k*-mer length, the hyperparameter tuning and parameter training steps were repeated with *k* values of 6–12.

To estimate the importance of each feature in the annotation models, the gain metric was extracted from each XGBoost model.

The output of each antimicrobial sub-model was a continuous value on a log_2_ scale. Therefore, predictions were first transformed using the power of two, and then censored to the nearest doubling dilution to allow comparison with the reference MICs.

### Progressive Genome Segment Enhancement (PGSE) algorithm

The PGSE algorithm started by generating *k*-mer counts and produced an initial feature space, *F*. For each round of PGSE, *F* was partitioned equally into *m* feature sub-spaces *F*_*i*_, where *i*∈{1,2,…,*m*} and

1 $$m=\max \left(\lfloor \frac{\left|F\right|}{5000}\rfloor ,1\right),$$

where |*F*| is the number of features in *F* and $\lfloor * \rfloor$ is the floor function. Therefore,

2 $$F=\mathop{\bigcup ^{m}}\limits_{i=1}{F}_{i}$$

and each subspace *F*_*i*_ contained approximately 5000 features. Each partition *F*_*i*_ was used to train a separate XGBoost model, each with a maximum tree depth of 3, with a learning rate of 0.001; the rest of the XGBoost parameters were kept to their default values. The feature selection process was as follows:

1. *Feature importance assignment:* Each feature *f*∈*F*_*i*_ in a subspace is assigned an importance score *s*(*f*) based on the XGBoost gain metric, which quantifies the contribution of the feature to the model’s predictive performance.
2. *Aggregation:* The feature importance scores across all subspaces *F*_*i*_ for *i* = 1,2,…,*m* are combined into a unified feature space *S*, where
  3 $$S=\{s\left({f}_{i}\right){\rm{| }}i=0,1,\ldots ,\left|F\right|\}.$$
3. *Ranking:* The features in *F* are ordered in descending order according to their importance scores *s*(*f*), forming a ranked list
  4 $${F}_{{\rm{sorted}}}=\{{f}_{1},{f}_{2},\ldots ,{f}_{\left|F\right|}\},$$
  such that
  5 $$s\left({f}_{1}\right)\ge s\left({f}_{2}\right)\ge \cdots \ge s\left({f}_{\left|F\right|}\right).$$
4. *Selection*: The top 10,000 features with the highest importance scores are selected from *F*_sorted_, denoted as

6 $${F}_{{\rm{top}}}=\{{f}_{1},{f}_{2},\ldots ,{f}_{10,000}\},$$

and carried forward to the next step of the PGSE algorithm.

Next, redundant segments from *F*_top_ were removed. For example, for two segments, *α* and *β*, where *α* = *ATTGCTAGCGTT* and *β* = *TGCTAGCG*, *β* is a subsequence of *α*. For any such pairs of segments, the segment with the higher importance score was retained— the other was discarded. Each retained segment was then extended by pre-pending and appending all nucleotide permutations. The length of the new segments was determined by the extension hyperparameter *p*. Given a segment *ω*∈*N*^*j*^ (where *N* is the set of all nucleotides {*A,C,G,T*} and *j* is the length of *ω*), PGSE iteratively generated extended segments that had lengths from *j*+1 to *j*+p. This was achieved by iteratively applying the following function over *l*, where *l*∈{1,2,…,*p*}:

7 $$O\left(\omega ,l\right)=\mathop{\bigcup }\limits_{n\in {N}^{l}}\mathop{\bigcup ^{l}}\limits_{i=0}\left\{n\left[1..i\right]\cdot \omega \cdot n\left[i+1..l\right]\right\},$$

where,

- *N*^*l*^ is the set of all extension strings of length *l*, e.g., *N*^2^ = {*AA,AC,AG*,...,*TC,TG,TT*};
- *n*∈*N*^*l*^ represents a single extension string, e.g., *n* = *AC* when *l* = 2;
- *n*[1..*i*] is the prefix of *n* of length *i*, such that *n*[1..0] is a zero-length extension and *n*[1..1] = *A* for *n* = *AC* and *i* = 1;
- *n*[*i*+1..*l*] is the suffix of *n* from position *i*+1 to *l*, such that *n*[0+1..2] = *AC* and *n*[1+1..2] = *C* for *n* = *AC* and *i* = 1;
- *ω* is the original sequence;
⋅ is the segment concatenation operator.

Ultimately, $\hat{O}$ was generated, which was the union of each output from *O*:

8 $$\hat{O}\left(\omega ,p\right)=\mathop{\bigcup ^{p}}\limits_{l=1}\{O\left(\omega ,l\right)\}$$

For any starting segment *ω*, the number of members of set $\hat{O}$ was always:

9 $$\left|\hat{O}\right|=\mathop{\sum }\limits_{l=1}^{p}{\left|N\right|}^{l}\times \left(l+1\right)$$

*For example*, let *ω* = *ATACCG* (*j* = 6) and *p* = 2.

*First iteration* (*l* = 1)

The set of all extension strings of length 1 is *N*^1^ = {*A,C,G,T*}. Using Eq. 8, the extended sequences are:

10 $$O\left(\omega ,1\right)=\mathop{\bigcup }\limits_{n\in {N}^{1}}\mathop{\bigcup ^{1}}\limits_{i=0}\left\{n\left[1..i\right]\cdot \omega \cdot n\left[i+1..1\right]\right\}$$

Simplifying, there are 8 sequences of length 7:

11 $$O\left(\omega ,1\right)=\{A\omega ,C\omega ,G\omega ,T\omega ,\omega A,\omega C,\omega G,\omega T\}$$

*Second iteration* (*l* = 2)

The set of all extension strings of length 2 is *N*^2^, which has 4^2^ = 16 elements. For each *n*∈*N*^2^ and *i*∈{0,1,2}, we construct the extended sequences:

12 $$O\left(\omega ,2\right)=\mathop{\bigcup }\limits_{n\in {N}^{2}}\mathop{\bigcup ^{2}}\limits_{i=0}\left\{n\left[1..i\right]\cdot \omega \cdot n\left[i+1..2\right]\right\}$$

This results in 16 × 3 = 48 sequences with length 8, such as:

13 $$\begin{array}{l}\{\\ AA\omega ,AC\omega ,AG\omega ,...,TC\omega ,TG\omega ,TT\omega ,\\ A\omega A,A\omega C,A\omega G,...,T\omega C,T\omega G,T\omega T,\\ \omega AA,\omega AC,\omega AG,...,\omega TC,\omega TG,\omega TT\\ \}\end{array}$$

To avoid repetitive segment extension, segments that had undergone extension were not re-extended on subsequent rounds of PGSE.

Finally, the algorithm generated counts of the retained and extended segments in each genome and used this for the updated feature space, *F*. The Aho–Corasick algorithm^49^, modified to only count canonical substrings, was used for fast multi-substring matching. The PGSE algorithm now returned to the feature partitioning step for another round. The algorithm was considered to have converged when the segment extension step did not propose any new extensions (i.e. when all the extended segments in the latest round had a lower importance than the previous round). After converging, PGSE produced a trained XGBoost model and a feature set of variable-length genome segments that predicted MIC.

The PGSE algorithm had the following hyperparameters: (1) the initial *k*-mer length; and (2) *p*, the extension hyperparameter. Three different values of *k* (6, 8, and 10) were explored for the initial *k*-mer length. The extension hyperparameter *p* was kept constant at 2.

### Internal validation

Model performance was first evaluated on the study dataset using cross-validation; no external data was used at this stage. Prior to the next evaluation steps, the predicted MICs from each cross-validation fold were combined into a single dataset for each sub-model. In total, 30 sets of predicted MICs were generated (10 antimicrobial sub-models for the *k*-mer model, 10 antimicrobial sub-models for the annotations model, 10 antimicrobial sub-models for the PGSE model).

The predicted MICs were compared to the reference MICs (defined as the MICs determined by agar dilution) using the following metrics: (1) essential agreement (EA), defined as the proportion of predicted MICs that were within ± 1 dilution of the true MIC; (2) categorical agreement (CA).

Categorical agreement between predicted and reference MICs was assessed by firstly categorising MICs into “S”, “I”, or “R” using EUCAST breakpoints (2023 version)^13^. For agents where no EUCAST breakpoints were available, epidemiological cut-off values (ECOFFs) were used^50^. Categorical agreement was used to calculate rates of minor error (mE, “I” on one method; “S” or “R” on the other method), major error (ME, prediction of “R”; “S” on the reference method), and very major error (vME, prediction of “S”; “R” on the reference method).

### External validation

To evaluate the generalisability of the models, an external dataset was generated by retrieving *E. coli* WGS data from the BV-BRC database (formerly known as PATRIC)^22^. Only genomes with available meta-data (MIC) for one or more of this study’s antimicrobials were included; 4755 *E. coli* genomes were available. The MIC values were changed to the nearest doubling dilution to allow comparison with the MICs generated in this study. Since five models were generated for each antimicrobial (one for each cross-validation fold), five predicted MICs were generated from each genome available in the external dataset. The median of the five MICs was used as the final predicted MIC.

Next, MIC values were converted into “S”, “I”, or “R”, again using EUCAST 2023 breakpoints^13^. These categorical values were then converted into binary values, where “S” was coded as 0, and “I” or “R” were coded as 1. This binary encoding allowed the cross-tabulation of predicted and reference MICs: (1) true positives (*TN*) where prediction was “I” / “R” and reference “I” / “R”; (2) true negatives (*TN*) where prediction was “S” and reference “S”; (3) false positive (*FP*) where prediction was “I” / “R” and reference “S”; and (4) false negative (*FN*) where prediction was “S” and reference “I” / “R”. To evaluate model performance, the F1 score, sensitivity, and specificity were calculated as follows:

14 $$F1=2\cdot \frac{{\rm{precision}}\cdot {\rm{recall}}}{{\rm{precision}}+{\rm{recall}}},$$

where

15 $${\rm{precision}}=\frac{{TP}}{{TP}+{FP}},$$

16 $${\rm{sensitivity}}={\rm{recall}}=\frac{{TP}}{{TP}+{FN}},$$

and

$${\rm{specificity}}=\frac{{TN}}{{TN}+{FP}}.$$

Cross-tabulation and F1 score calculation were performed using the caret R package (version 7.0-1)^51^. To quantify the performance of the models on unseen external data (i.e., generalisation), the F1 score was calculated for the internal (cross-validation) dataset and compared to the F1 score calculated on the external dataset. This process was repeated for each model type (*k*-mer, annotations, and PGSE) and for each antimicrobial sub-model. The difference between the internal and external dataset F1 scores was calculated as the generalisation gap — negative values indicate that the model performed better on the internal dataset than on the external dataset, while positive values indicate that the model performed better on the external dataset:

17 $${\rm{Generalisation\; gap}}({GG})={{\rm{F}}1}_{{\rm{internal}}}-{{\rm{F}}1}_{{\rm{external}}}.$$

The same process was repeated to calculate the generalisation gap for sensitivity and specificity.

### Statistical analysis

To evaluate the correlation between true MICs and predicted MICs, the Pearson correlation coefficient was calculated for each antimicrobial sub-model. The coefficient was calculated on log_2_ transformed and uncensored MICs (as described above); the correlation between true and predicted MIC was assumed to be linear.

### Computational environment and software

Models were trained and evaluated on a high-performance computing cluster; each node was equipped with 40 CPU cores and 384GB of RAM. To test the resource utilisation on a consumer computer, ceftazidime models were also trained and evaluated (using an 80% training and 20% validation split) on an isolated Windows Subsystem for Linux (WSL) with 60GB of RAM. The WSL ran on a consumer-grade computer equipped with an Intel Core i9-13950HX CPU and 64GB of RAM.

The R programming language was used for data processing and visualisation^52^. The AMR and MIC R packages were used to manipulate antimicrobial susceptibility results^48,53^. The PGSE algorithm was implemented in the Python programming language^54^; an R implementation is also available^52^.

## Supplementary information


Supplementary material


## Data Availability

The data used in this study are available under a CC-BY licence at: https://datacat.liverpool.ac.uk/id/eprint/3008^55^. The PGSE algorithm is available under an open source AGPL-3.0 licence at: https://github.com/yinzheng-zhong/PGSE. The WGS data for the strains used in this study are available on the NCBI GenBank Sequence Read Archive under the following accession number: PRJNA1297298. The code to generate the data used in this manuscript is available at: https://github.com/agerada/molecular_mic_analysis.

## References

[1] Ellington, M. J. et al. The role of whole genome sequencing in antimicrobial susceptibility testing of bacteria: report from the EUCAST Subcommittee. *Clin. Microbiol. Infect.* **23**, 2–22 (2017). 27890457 10.1016/j.cmi.2016.11.012

[2] Kim, J. I. et al. Machine learning for antimicrobial resistance prediction: current practice, limitations, and clinical perspective. *Clin. Microbiol. Rev.* e00179–21 10.1128/cmr.00179-21 (2022). PMC9491192 35612324

[3] Pataki, B. Á et al. Understanding and predicting ciprofloxacin minimum inhibitory concentration in Escherichia coli with machine learning. *Sci. Rep.* **10**, 15026 (2020). 32929164 10.1038/s41598-020-71693-5 PMC7490380

[4] Pal, A. & Mohanty, D. Machine learning-based approach for identification of new resistance associated mutations from whole genome sequences of *Mycobacterium Tuberculosis*. *Bioinform. Adv.* **5**, vbaf050 (2024). 10.1093/bioadv/vbaf050 PMC11930343 40125545

[5] Bradley, P. et al. Rapid antibiotic-resistance predictions from genome sequence data for Staphylococcus aureus and Mycobacterium tuberculosis. *Nat. Commun.* **6**, 10063 (2015). 26686880 10.1038/ncomms10063 PMC4703848

[6] Ren, Y. et al. Prediction of antimicrobial resistance based on whole-genome sequencing and machine learning. *Bioinformatics* **38**, 325–334 (2022). 34613360 10.1093/bioinformatics/btab681 PMC8722762

[7] Aytan-Aktug, D., Clausen, P. T. L. C., Bortolaia, V., Aarestrup, F. M. & Lund, O. Prediction of acquired antimicrobial resistance for multiple bacterial species using neural networks. *mSystems* **5**, e00774–19 (2020). 31964771 10.1128/mSystems.00774-19 PMC6977075

[8] Nguyen, M. et al. Developing an in silico minimum inhibitory concentration panel test for Klebsiella pneumoniae. *Sci. Rep.* **8**, 421 (2018). 29323230 10.1038/s41598-017-18972-w PMC5765115

[9] Nguyen, M. et al. Using machine learning to predict antimicrobial MICs and associated genomic features for nontyphoidal *Salmonella*. *J. Clin. Microbiol.* **57**, e01260–18 (2019). 30333126 10.1128/JCM.01260-18 PMC6355527

[10] Gao, Y. et al. Machine learning and feature extraction for rapid antimicrobial resistance prediction of Acinetobacter baumannii from whole-genome sequencing data. *Front. Microbiol.* **14**, 1320312 (2024). 38274740 10.3389/fmicb.2023.1320312 PMC10808480

[11] EUCAST. *The European Committee on Antimicrobial Susceptibility Testing. Breakpoint Tables for Interpretation of MICs and Zone Diameters*. (2022).

[12] CLSI. *Performance Standards for Antimicrobial Susceptibility Testing*. (Wayne, PA, 2020).

[13] The European Committee on Antimicrobial Susceptibility Testing. *EUCAST Breakpoint Tables* (2023).

[14] Krawczyk, B. Learning from imbalanced data: open challenges and future directions. *Prog. Artif. Intell.* **5**, 221–232 (2016).

[15] Ho, P.-L. et al. Genetic identity of aminoglycoside-resistance genes in Escherichia coli isolates from human and animal sources. *J. Med. Microbiol.* **59**, 702–707 (2010). 20185552 10.1099/jmm.0.015032-0

[16] Yan, J.-J. et al. Plasmid-mediated 16S rRNA methylases conferring high-level aminoglycoside resistance in Escherichia coli and Klebsiella pneumoniae isolates from two Taiwanese hospitals. *J. Antimicrob. Chemother.* **54**, 1007–1012 (2004). 15486082 10.1093/jac/dkh455

[17] Škunca, N. et al. Phyletic profiling with cliques of orthologs is enhanced by signatures of paralogy relationships. *PLoS Comput. Biol.* **9**, e1002852 (2013). 23308060 10.1371/journal.pcbi.1002852 PMC3536626

[18] Zhao, W.-H. & Hu, Z.-Q. Epidemiology and genetics of CTX-M extended-spectrum β-lactamases in Gram-negative bacteria. *Crit. Rev. Microbiol.* **39**, 79–101 (2013). 22697133 10.3109/1040841X.2012.691460 PMC4086240

[19] Hendrickx, A. P. A. et al. Bla OXA-48-like genome architecture among carbapenemase-producing Escherichia coli and Klebsiella pneumoniae in the Netherlands. *Micro. Genom.* **7**, 000512 (2021). 10.1099/mgen.0.000512 PMC8209719 33961543

[20] Manyahi, J. et al. First identification of bla NDM-5 producing Escherichia coli from neonates and a HIV infected adult in Tanzania. *J. Med. Microbiol.* **71**, 001513 (2022). 35225760 10.1099/jmm.0.001513 PMC8941953

[21] Chen, T. & Guestrin, C. XGBoost: a scalable tree boosting system. In *Proceedings of the 22nd ACM SIGKDD International Conference on Knowledge Discovery and Data Mining* 785–794 (ACM, San Francisco California USA, 2016). 10.1145/2939672.2939785.

[22] Olson, R. D. et al. Introducing the Bacterial and Viral Bioinformatics Resource Center (BV-BRC): a resource combining PATRIC, IRD and ViPR. *Nucleic Acids Res* **51**, D678–D689 (2023). 36350631 10.1093/nar/gkac1003 PMC9825582

[23] Eyre, D. W. et al. WGS to predict antibiotic MICs for Neisseria gonorrhoeae. *J. Antimicrobial. Chemother.* **72**, 1937–1947 (2017). 10.1093/jac/dkx067 PMC5890716 28333355

[24] Nguyen, Q. H. et al. eMIC-AntiKP: estimating minimum inhibitory concentrations of antibiotics towards Klebsiella pneumoniae using deep learning. *Comput. Struct. Biotechnol. J.* **21**, 751–757 (2023). 36659924 10.1016/j.csbj.2022.12.041 PMC9827358

[25] Ryu, B., Jeon, W. & Kim, D. Integrating genomic and molecular data to predict antimicrobial minimum inhibitory concentration in Klebsiella pneumoniae. *Sci. Rep.* **14**, 25951 (2024). 39472617 10.1038/s41598-024-75973-2 PMC11522393

[26] Moeckel, C. et al. A survey of k-mer methods and applications in bioinformatics. *Comput. Struct. Biotechnol. J.* **23**, 2289–2303 (2024). 38840832 10.1016/j.csbj.2024.05.025 PMC11152613

[27] Asnicar, F., Thomas, A. M., Passerini, A., Waldron, L. & Segata, N. Machine learning for microbiologists. *Nat. Rev. Microbiol.* **22**, 191–205 (2024). 37968359 10.1038/s41579-023-00984-1 PMC11980903

[28] Mouton, J. W., Meletiadis, J., Voss, A. & Turnidge, J. Variation of MIC measurements: the contribution of strain and laboratory variability to measurement precision. *J. Antimicrob. Chemother.* **73**, 2374–2379 (2018). 30137390 10.1093/jac/dky232

[29] African Society of Laboratory Medicine Mapping AMR & AMU Partnership. *Incomplete Antimicrobial Resistance (AMR) Data in Africa: The Crisis Within The Crisis*. https://aslm.org/wp-content/uploads/2022/09/ASLM_MAAP-Policy-Brief_Embargoed-until-15-Sept-6AM-GMT.pdf?x60402 (2022).

[30] *Antimicrobial Resistance Diagnostic Initiative: Strengthening Bacteriology and Mycology Diagnostic Capacity, Laboratory Systems and Service Delivery* (World Health Organization, Geneva, 2024).

[31] United Nations. UN General Assembly High-Level Meeting on antimicrobial resistance 2024. https://www.who.int/news-room/events/detail/2024/09/26/default-calendar/un-general-assembly-high-level-meeting-on-antimicrobial-resistance-2024.

[32] Howard, A. et al. Personalised antimicrobial susceptibility testing with clinical prediction modelling informs appropriate antibiotic use. *Nat. Commun.* **15**, 9924 (2024). 39572574 10.1038/s41467-024-54192-3 PMC11582675

[33] Jaillard, M. et al. A fast and agnostic method for bacterial genome-wide association studies: bridging the gap between k-mers and genetic events. *PLoS Genet.* **14**, e1007758 (2018). 30419019 10.1371/journal.pgen.1007758 PMC6258240

[34] Jaillard, M., Palmieri, M., Van Belkum, A. & Mahé, P. Interpreting *k* -mer–based signatures for antibiotic resistance prediction. *GigaScience* **9**, giaa110 (2020). 33068113 10.1093/gigascience/giaa110 PMC7568433

[35] Humphries, R. M. et al. Machine-learning model for prediction of cefepime susceptibility in Escherichia coli from whole-genome sequencing data. *J. Clin. Microbiol.* **61**, e01431–22 (2023). 36840604 10.1128/jcm.01431-22 PMC10035297

[36] Wiegand, I., Hilpert, K. & Hancock, R. E. W. Agar and broth dilution methods to determine the minimal inhibitory concentration (MIC) of antimicrobial substances. *Nat. Protoc.* **3**, 163–175 (2008). 18274517 10.1038/nprot.2007.521

[37] Gerada, A., Harper, N., Howard, A. & Hope, W. AIgarMIC: A Python package for automated interpretationof agar dilution minimum inhibitory concentration assays. *JOSS* **9**, 6826 (2024).

[38] Gerada, A., Harper, N., Howard, A., Reza, N. & Hope, W. Determination of minimum inhibitory concentrations using machine-learning-assisted agar dilution. *Microbiol. Spectr.* **12**, e04209–e04223 (2024). 38517194 10.1128/spectrum.04209-23 PMC11064640

[39] Martin, M. Cutadapt removes adapter sequences from high-throughput sequencing reads. *EMBnet J.* **17**, 10 (2011).

[40] Bankevich, A. et al. SPAdes: a new genome assembly algorithm and its applications to single-cell sequencing. *J. Comput. Biol.* **19**, 455–477 (2012). 22506599 10.1089/cmb.2012.0021 PMC3342519

[41] Feldgarden, M. et al. AMRFinderPlus and the Reference Gene Catalog facilitate examination of the genomic links among antimicrobial resistance, stress response, and virulence. *Sci. Rep.* **11**, 12728 (2021). 34135355 10.1038/s41598-021-91456-0 PMC8208984

[42] Feldgarden, M. et al. Validating the AMRFinder tool and resistance gene database by using antimicrobial resistance genotype-phenotype correlations in a collection of isolates. *Antimicrob. Agents Chemother.* **63**, e00483–19 (2019). 31427293 10.1128/AAC.00483-19 PMC6811410

[43] Ondov, B. D. et al. Mash: Fast genome and metagenome distance estimation using MinHash. *Genome Biol.* **17**, 132 (2016). 27323842 10.1186/s13059-016-0997-x PMC4915045

[44] Letunic, I. & Bork, P. Interactive Tree of Life (iTOL) v6: recent updates to the phylogenetic tree display and annotation tool. *Nucleic Acids Res.* **52**, W78–W82 (2024). 38613393 10.1093/nar/gkae268 PMC11223838

[45] Grant, J. R. et al. Proksee: in-depth characterization and visualization of bacterial genomes. *Nucleic Acids Res.* **51**, W484–W492 (2023). 37140037 10.1093/nar/gkad326 PMC10320063

[46] Seemann, T. Prokka: Rapid prokaryotic genome annotation. *Bioinformatics* **30**, 2068–2069 (2014). 24642063 10.1093/bioinformatics/btu153

[47] Alcock, B. P. et al. CARD 2023: Expanded curation, support for machine learning, and resistome prediction at the Comprehensive Antibiotic Resistance Database. *Nucleic Acids Res.* **51**, D690–D699 (2023). 36263822 10.1093/nar/gkac920 PMC9825576

[48] Berends, M. S. et al. AMR: an R package for working with antimicrobial resistance data. *J. Stat. Soft.* **104**, 1–31 (2022).

[49] Aho, A. V. & Corasick, M. J. Efficient string matching: An aid to bibliographic search. *Commun. ACM* **18**, 333–340 (1975).

[50] Kahlmeter, G. & Turnidge, J. How to: ECOFFs—the why, the how, and the don’ts of EUCAST epidemiological cutoff values. *Clin. Microbiol. Infect.* **28**, 952–954 (2022). 35218980 10.1016/j.cmi.2022.02.024

[51] Kuhn, M. Building predictive models in R using the Caret package. *J. Stat. Soft.* **28**, 1–26 (2008).

[52] *R Core Team. R: A Language and Environment for Statistical Computing* (R Foundation for Statistical Computing, 2022).

[53] Gerada, A. *MIC: Analysis of Antimicrobial Minimum Inhibitory Concentration Data. 1.0.2 Comprehensive R Archive Network* (2025).

[54] Van Rossum, G. & Drake Jr, F. L. *Python Reference Manual* (Centrum voor Wiskunde en Informatica Amsterdam, 1995).

[55] Gerada, A. et al. Whole genome sequencing data for 762 clinical Escherichia coli strains with antimicrobial susceptibility meta data. University of Liverpool 10.17638/DATACAT.LIVERPOOL.AC.UK/3008 (2025).

