# Supplementary material for: Prediction of antimicrobial minimum inhibitory concentration from bacterial genomes using a scalable and interpretable machine learning approach
Source: NPJ Antimicrob Resist. 2026 May 26;4:51. doi: 10.1038/s44259-026-00217-4 (PMC13530227; doi:10.1038/s44259-026-00217-4)
Supplement: Supplementary file 1 [file 44259_2026_217_MOESM1_ESM.pdf]

Supplementary material

Antimicrobials with imbalanced data

This section contains Figures and Tables for the following antimicrobials that had imbalanced data, and were therefore not included in the main analysis:

- amoxicillin,
- amoxicillin-clavulanic acid,
- amikacin,
- chloramphenicol,
- tigecycline.

These figures and tables are otherwise unchanged from those in the main text.

Table S1: Essential and categorical agreement rates for k-mer ($k = 10$), annotation (Annot.) and PGSE (starting $k = 6$) models. Predictions were considered to be in essential agreement if the predicted MIC was within one doubling dilution of the reference MIC. For categorical agreement, minor error (me) was defined as I on one method and S/R on the other method; major error (ME) was defined as a prediction of R and S on the reference method; and very major error (vME) was defined as a prediction of S and R on the reference method

Antimicrobial	n MICs	Resistant n (%)	Model	EA n (%)[1]	mE n (%)[2]	ME n (%)[3]	vME n (%)[4]	r[5]
Amoxicillin-clavulanic acid	450	391 (86.9)	*k*-mer	352 (78.2)	0 (0)	54 (12)	4 (0.9)	0.43
			Annot.	369 (82)	0 (0)	56 (12.4)	1 (0.2)	0.62
			PGSE	376 (83.6)	0 (0)	58 (12.9)	1 (0.2)	0.38
Amikacin	717	38 (5.3)	*k*-mer	665 (92.7)	0 (0)	0 (0)	38 (5.3)	0.50
			Annot.	666 (92.9)	0 (0)	0 (0)	38 (5.3)	0.50
			PGSE	658 (91.8)	0 (0)	0 (0)	38 (5.3)	0.48
Amoxicillin	544	478 (87.9)	*k*-mer	436 (80.1)	0 (0)	62 (11.4)	0 (0)	0.40
			Annot.	458 (84.2)	0 (0)	42 (7.7)	10 (1.8)	0.62
			PGSE	464 (85.3)	0 (0)	66 (12.1)	0 (0)	0.30
Chloramphenicol	709	48 (6.8)	*k*-mer	594 (83.8)	0 (0)	0 (0)	40 (5.6)	0.41
			Annot.	607 (85.6)	0 (0)	6 (0.8)	27 (3.8)	0.58
			PGSE	599 (84.5)	0 (0)	2 (0.3)	37 (5.2)	0.43
Tigecycline	271	1 (0.4)	*k*-mer	257 (94.8)	0 (0)	0 (0)	1 (0.4)	0.33
			Annot.	254 (93.7)	0 (0)	0 (0)	1 (0.4)	0.27
			PGSE	260 (95.9)	0 (0)	0 (0)	1 (0.4)	0.22

[1]Essential agreement (EA); [2]minor error (mE); [3]major error (ME); [4]very major error (vME); [5]Pearson correlation coefficient (r);

Table S2: Top three genes with highest feature importance for each annotation model.

Gene symbol	Feature importance (%)	Cumulative feature importance (%)	Resistance activity
**Amoxicillin/clavulanic acid**			
blaTEM-1	17.7	17.7	Beta-lactam
blaCTX-M-15	14.5	32.3	Cephalosporin
blaOXA-48	7.7	40.0	Carbapenem
**Amikacin**			
aac(6')-Ib-cr5	35.4	35.4	Amikacin, kanamycin, quinolone, tobramycin
catB3	11.1	46.6	Chloramphenicol
gyrA_D87N	7.9	54.4	Quinolone
**Amoxicillin**			
blaOXA-48	24.1	24.1	Carbapenem
blaCTX-M-15	9.8	33.8	Cephalosporin
blaTEM-1	9.0	42.8	Beta-lactam
**Chloramphenicol**			
catA1	41.2	41.2	Chloramphenicol
floR	40.3	81.5	Chloramphenicol, florfenicol
tet(A)	2.8	84.2	Tetracycline
**Tigecycline**			
tet(A)	30.3	30.3	Tetracycline
blaOXA-48	9.1	39.5	Carbapenem
blaTEM-1	3.1	42.5	Beta-lactam

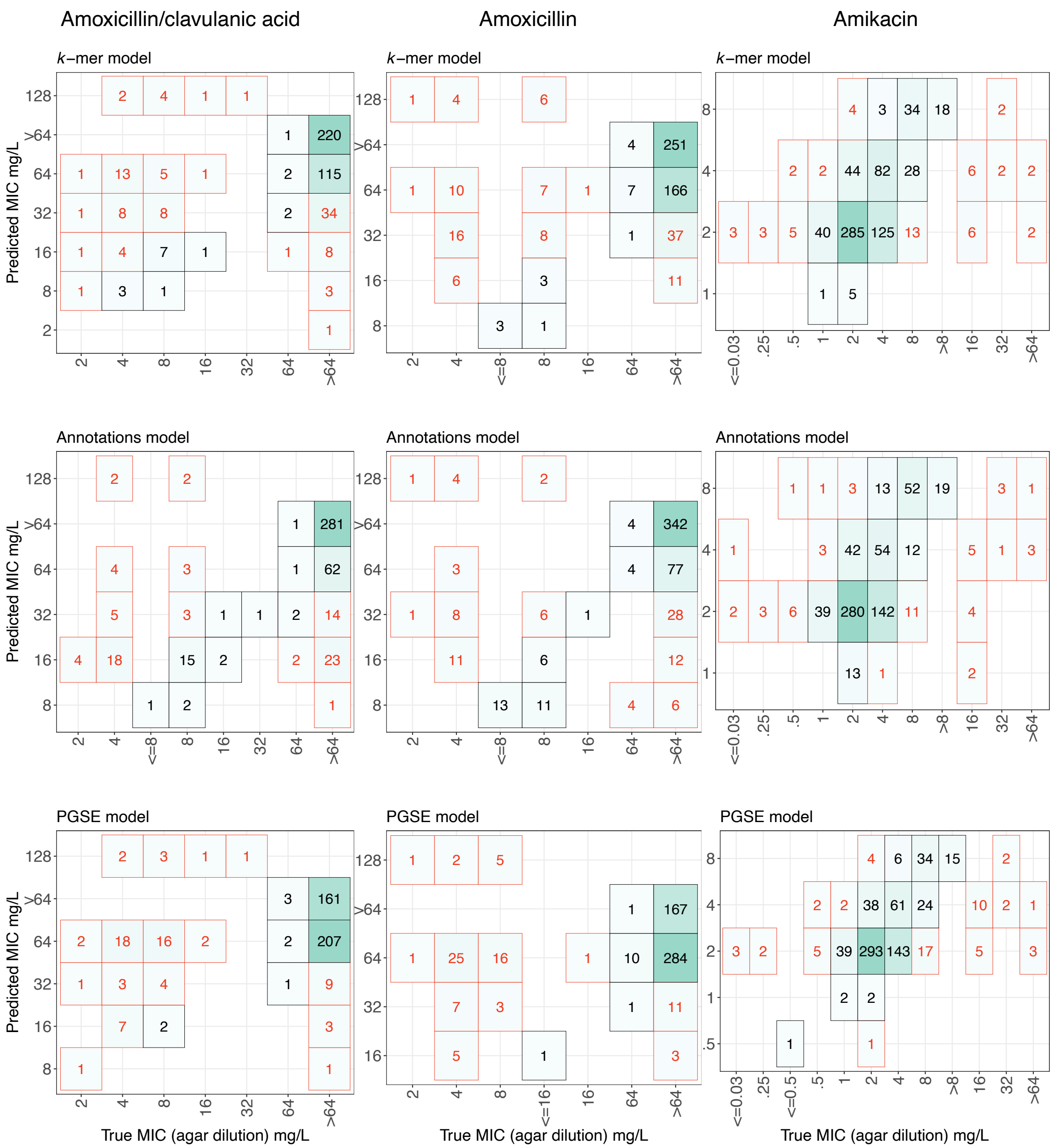

Figure S1: Predicted MICs from amoxicillin-clavulanic acid, amoxicillin, and amikacin models, using *k*-mer, annotations, and PGSE machine learning sub-models evaluated against true MIC, measured using agar dilution.

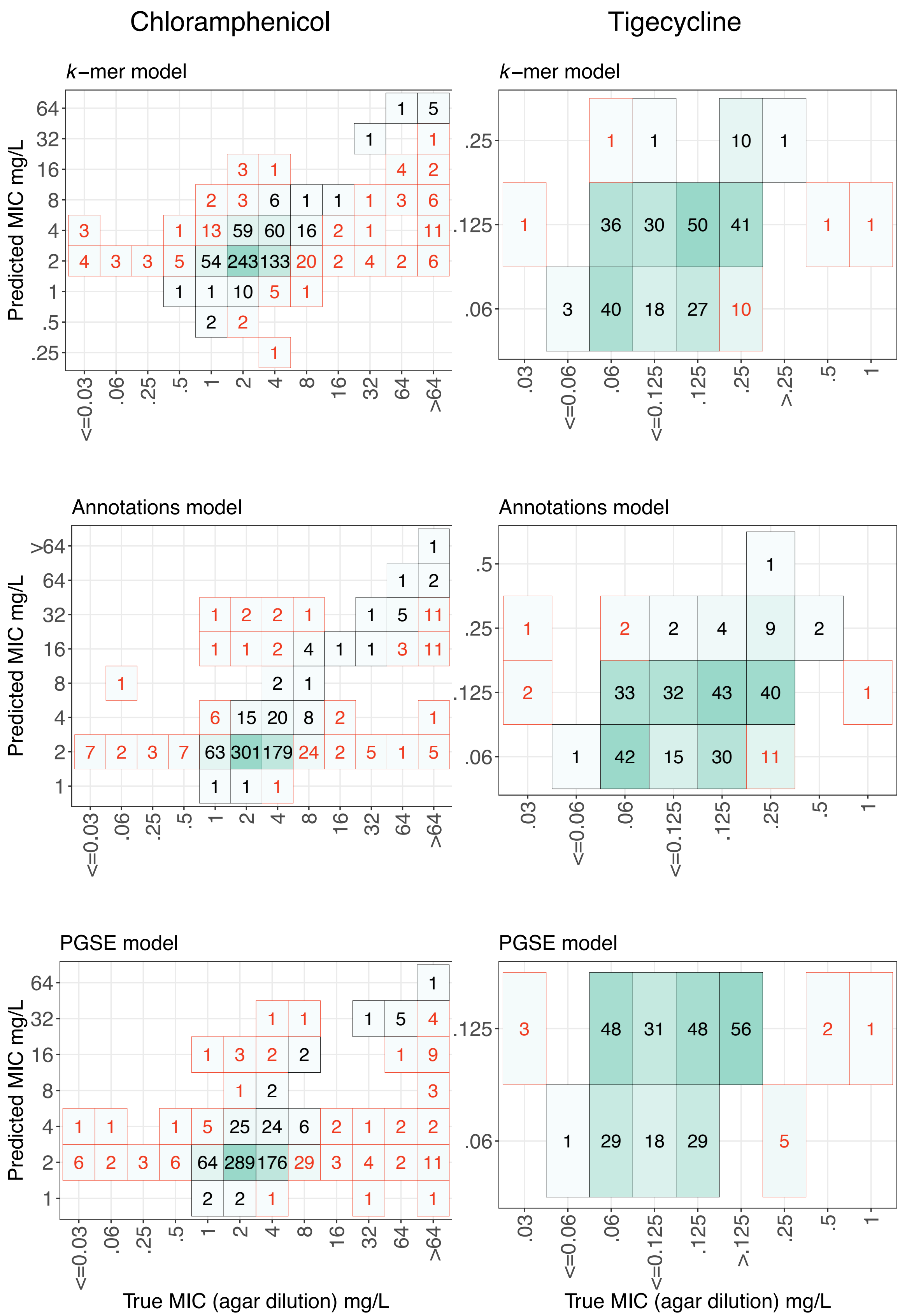

Figure S2: Predicted MICs from chloramphenicol and tigecycline models, using k-mer, annotations, and PGSE machine learning sub-models evaluated against true MIC, measured using agar dilution.

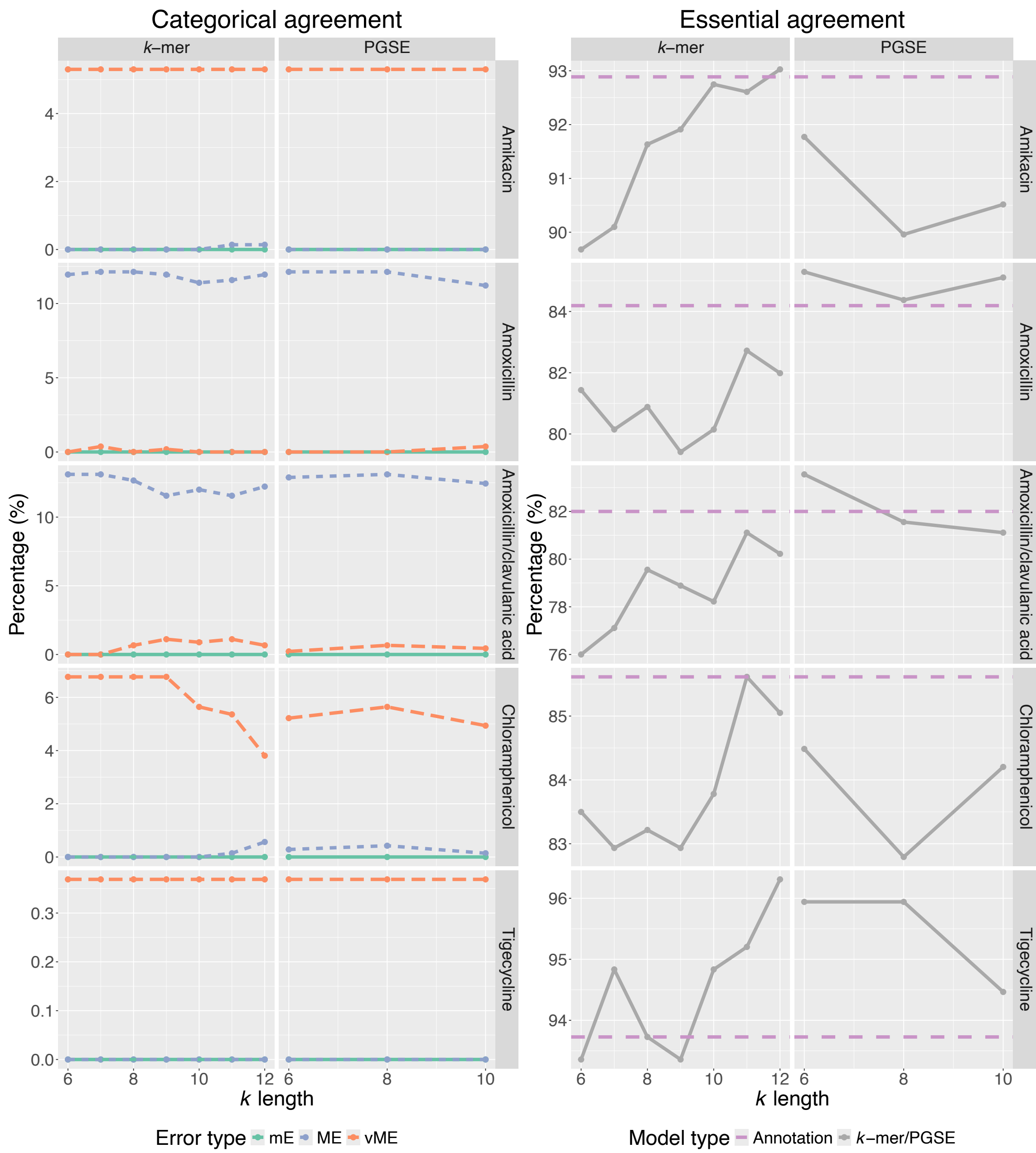

Figure S3: Essential agreement and error rates for the k-mer models across different lengths of k, compared to PGSE with different starting values for k; mE = minor error ("I" on one method; "S" or "R" on the other method); ME = major error (prediction of "R"; "S" on the reference method); and vME = very major error (prediction of "S"; "R" on the reference method).

Additional analyses

In this section, all results are pooled across the ten antimicrobials, including those with imbalanced data.

Table S3: Essential agreement rates pooled across all ten antimicrobials for *k*-mer, annotation, and PGSE models. This table includes antimicrobials with imbalanced data, in addition to those presented in the main text.

Antimicrobial	k-mer (%)	Annotations (%)	PGSE (%)
Amoxicillin/clavulanic acid	78.2	82.0	83.6
Amikacin	92.7	92.9	91.8
Amoxicillin	80.1	84.2	85.3
Ceftazidime	57.6	68.1	62.8
Chloramphenicol	83.8	85.6	84.5
Ciprofloxacin	65.4	83.6	75.5
Cefepime	58.7	64.3	63.6
Gentamicin	78.6	84.8	82.1
Meropenem	87.2	87.8	88.2
Tigecycline	94.8	93.7	95.9
**All (pooled)**	**76.3**	**81.8**	**80.0**

Table S4: Distributions of carbapenemase genes for both the external and internal datasets, with median meropenem MICs for genomes containing each gene.

Gene	n (%)	Median meropenem MIC (mg/L)
**External (n=4733)**		
blaIMP	21 (0.4%)	8.00
blaKPC	34 (0.7%)	4.00
blaNDM	45 (1%)	8.00
blaOXA-48	2 (0%)	0.50
blaVIM	2 (0%)	2.50
**Internal (n=762)**		
blaIMP	2 (0.3%)	3.00
blaKPC	4 (0.5%)	2.50
blaNDM	15 (2%)	16.00
blaOXA-48	140 (18.4%)	0.50
blaVIM	4 (0.5%)	0.75
